# Enol-mediated delivery of H_2_Se from γ-keto selenides: mechanistic insight and evaluation†

**DOI:** 10.1039/d2sc03533b

**Published:** 2022-10-19

**Authors:** Rynne A. Hankins, Molly E. Carter, Changlei Zhu, Chen Chen, John C. Lukesh

**Affiliations:** Department of Chemistry, Wake Forest University Wake Downtown Campus Winston-Salem NC 27101 USA lukeshjc@wfu.edu

## Abstract

Like hydrogen sulfide (H_2_S), its chalcogen congener, hydrogen selenide (H_2_Se), is an emerging molecule of interest given its endogenous expression and purported biological activity. However, unlike H_2_S, detailed investigations into the chemical biology of H_2_Se are limited and little is known about its innate physiological functions, cellular targets, and therapeutic potential. The obscurity surrounding these fundamental questions is largely due to a lack of small molecule donors that can effectively increase the bioavailability of H_2_Se through their continuous liberation of the transient biomolecule under physiologically relevant conditions. Driven by this unmet demand for H_2_Se-releasing moieties, we report that γ-keto selenides provide a useful platform for H_2_Se donation *via* an α-deprotonation/β-elimination pathway that is highly dependent on both pH and alpha proton acidity. These attributes afforded a small library of donors with highly variable rates of release (higher alpha proton acidity = faster selenide liberation), which is accelerated under neutral to slightly basic conditions—a feature that is unique and complimentary to previously reported H_2_Se donors. We also demonstrate the impressive anticancer activity of γ-keto selenides in both HeLa and HCT116 cells in culture, which is likely to stimulate additional interest and research into the biological activity and anticancer effects of H_2_Se. Collectively, these results indicate that γ-keto selenides provide a highly versatile and effective framework for H_2_Se donation.

## Abstract 2

Like hydrogen sulfide (H_2_S), its chalcogen congener, hydrogen selenide (H_2_Se), is an emerging biomolecule of interest. Therefore, small molecule donors that effectively increase its bioavailability are desired to further probe H_2_Se chemical biology.

## Graphical abstract

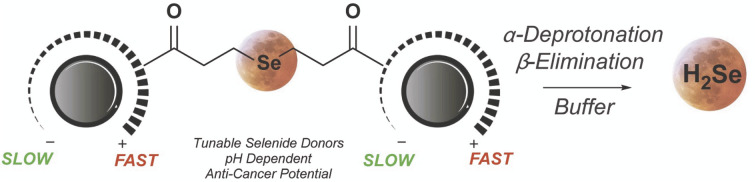

## Introduction

Gasotransmitters are a small family of endogenously expressed, gaseous signaling molecules that include nitric oxide (NO), carbon monoxide (CO), and hydrogen sulfide (H_2_S).^1–3^ For admittance into this select group, specific criteria must be met.^4^ The molecule must exist as a gas and be able to freely permeate cellular membranes. Moreover, its endogenous production should be enzymatically regulated, ensuring its ability to carry out specific biological functions at physiologically relevant concentrations. Lastly, it should elicit its physiological effects while acting on well-defined biological targets.

Hydrogen sulfide is the most recent member, with its inclusion first being suggested in the early 2000s.^4,5^ H_2_S is primarily produced in mammalian systems *via* the enzymatic catabolism of sulfur-containing amino acids.^6–9^ With particularly high expression levels in the central nervous system, endogenous H_2_S functions as a neuromodulator in the brain by enhancing NMDA receptor-mediated responses.^10,11^ It also plays an important role in vasodilation and blood pressure regulation through its activation of K_ATP_ channels.^12^ Additionally, H_2_S displays impressive antioxidative and cytoprotective effects stemming from its activation of the Keap1-Nrf-2 pathway, which leads to the upregulation of antioxidant enzymes.^13^ The underlying mechanism behind these, and other, H_2_S-mediated pharmacological effects is believed to involve the persulfidation of protein thiols (protein–SSH)—an important posttranslational modification that impacts protein structure and function.^14^

Similar to H_2_S, its chalcogen congener, hydrogen selenide (H_2_Se), is beginning to attract the attention of the scientific community as an endogenously expressed, biologically active gas. Sulfur and selenium possess similar physical and chemical properties. However, given its increased size and polarizability, reactions involving selenium tend to be significantly faster than analogous reactions involving sulfur.^15–17^ Therefore, it is conceivable that H_2_Se may promote comparable protein modifications (protein–SSeH) that influence cellular activity, even at significantly lower concentrations.

**Fig. 1:**
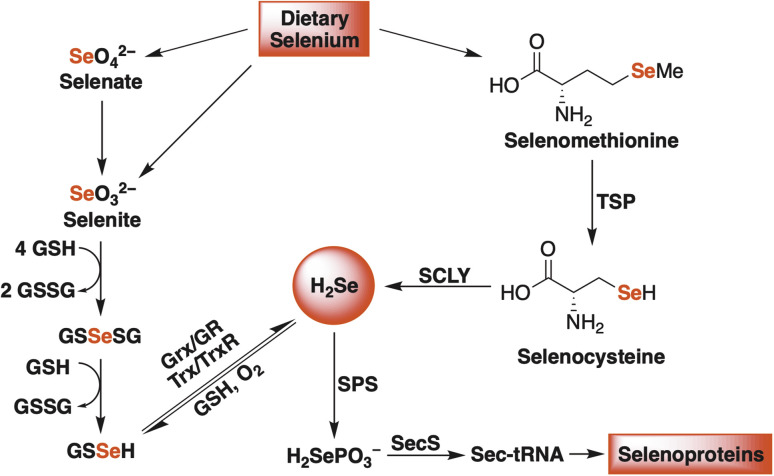
A simplified schematic of endogenous H_2_Se production in mammalian systems. Glutathione (GSH), glutathione disulfide (GSSG), glutaredoxin (Grx), glutathione reductase (GR), thioredoxin (Trx), thioredoxin reductase (TrxR), transsulfuration pathway (TSP), selenocysteine β-lyase (SCLY), selenophosphate synthetase (SPS), and selenocysteine synthase (SecS).

Hydrogen selenide is chiefly produced from the reduction of dietary inorganic selenium salts (selenate and selenite) and from the enzymatic breakdown of selenium-containing amino acids (selenomethionine) (Fig. 1).^18–20^ Once formed, H_2_Se is used to generate other selenium-containing biomolecules, including important redox-active proteins, like thioredoxin reductase (TrxR) and glutathione peroxidase (GPx), that rely on selenocysteine, nature's 21st amino acid, to maintain cellular redox homeostasis.^15,21^ Therefore, given its gaseous nature (in its diprotic form), its bioactivity, and the strict enzymatic control over its biosynthesis, H_2_Se already satisfies several gasotransmitter requirements, sparking some to suggest its inclusion as the fourth official gaseous signaling molecule in mammalian systems.^22^ While there is some evidence to support this claim, a clear description of its redox biochemistry remains poorly defined.

In many respects, H_2_Se is perceived similarly to how H_2_S was at the turn of the 20th century. Historically dismissed as a toxic and foul-smelling gas,^23^ in the past two decades the immense physiological and pathophysiological effects of H_2_S were largely uncovered thanks to the introduction of small molecule donors that were used to modulate cellular concentrations by effectively mimicking the natural, enzymatic production of H_2_S over a prolonged period of time.^24–27^ Conversely, analogous donors for exploring the biological prevalence and significance of H_2_Se are extremely scarce, which is why its innate physiological functions, molecular targets, and participation in redox signaling remain a mystery.

**Fig. 2:**
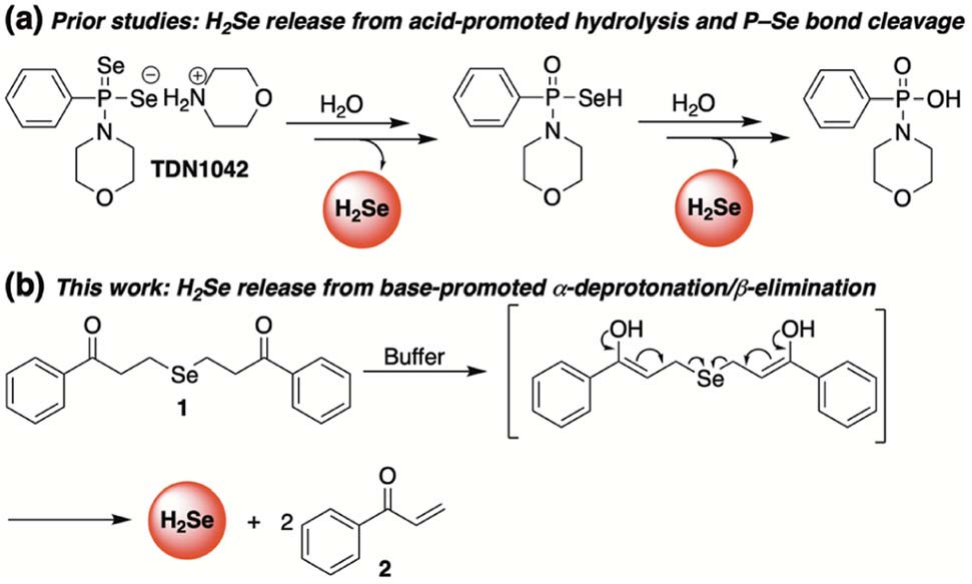
Complimentary H_2_Se donors promoted by acid (a) and base (b).

To meet this unmet demand, previously reported H_2_S donors are being reengineered to deliver H_2_Se in a controlled and sustained manner.^28–31^ Among the first to be examined was a hydrolysis-based donor reported by Pluth and co-workers.^29^ Taking inspiration from the widely studied H_2_S donor GYY4137,^32^ they generated an analogous slow-release donor that was shown to effectively liberate H_2_Se through hydrolytic P=Se bond cleavage in buffered solutions (TDN1042, Fig. 2). Consistent with a hydrolysis-based mechanism, the authors noted a significant increase in hydrolysis at more acidic pH values.

Complimentary to this initial report, we sought to cultivate a new class of slow-release donors that operate *via* an alternative mechanism such that their rate of H_2_Se delivery would be enhanced under neutral to slightly basic conditions. By applying lessons learned from earlier reported and successful H_2_S donors,^33^ we hypothesized that γ-keto selenides (1, Fig. 2) would meet this requirement by releasing H_2_Se through an α-deprotonation/β-elimination pathway that would be dependent on alpha proton acidity, thereby providing a series of related donors with highly tunable rates of H_2_Se release.

## Results discussion

Our investigations commenced by treating 3-chloropropiophenone with sodium selenide to afford 1 (Fig. 2) in moderate yield (see ESI† for details). Once in hand, we sought to investigate the propensity of 1 to release selenide in buffered water.

A current challenge in H_2_Se donor development is the lack of streamlined analytical techniques for effectively quantifying the delivery of hydrogen selenide over time. Since the chemical shift of the methylene protons of our donor (1) are easily distinguishable from those of the enone byproduct (2), we reasoned that ^1^H NMR would provide a convenient analytical method for monitoring the conversion of 1 to 2 and, thus, the release of selenide.

**Fig. 3:**
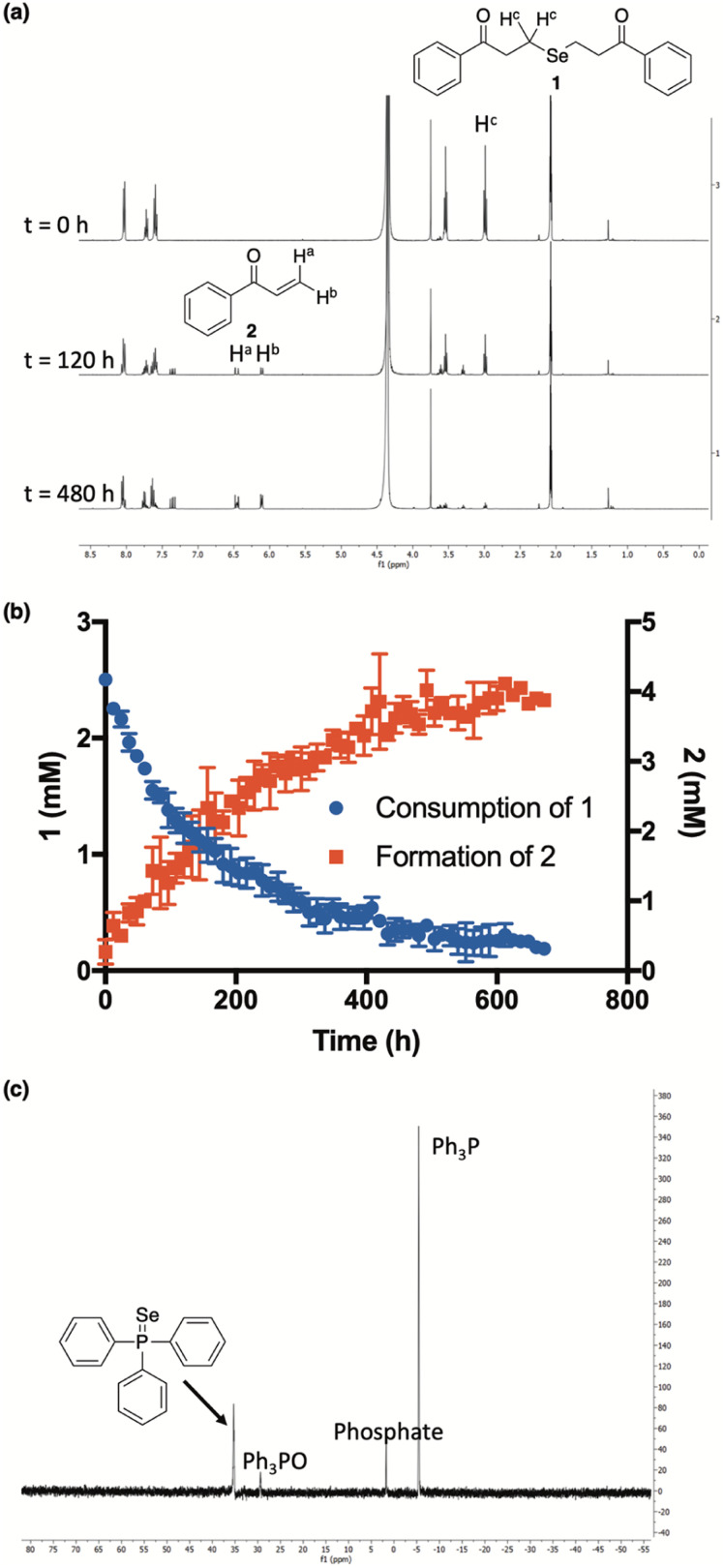
(a and b) Time-course for the conversion of ketone 1 (2.5 mM) into enone 2 at ambient temperature and in a 1 : 1 mixture of CD_3_CN and deuterated phosphate buffer (50 mM, pD 7.4). 1,4-Dioxane was used as an internal standard to determine the concentration of 1 and 2 at different time points. All reactions were performed in triplicate and plotted as the mean ± STDEV. (c) Resultant ^31^P NMR spectrum from the reaction between triphenylphosphine (PPh_3_) and precipitated selenium (Se^0^) from the decomposition of 1 in solution.

Indeed, in a 1 : 1 mixture of CD_3_CN and deuterated phosphate buffer (50 mM, pD 7.4), the clean conversion of 1 to 2 was observed by ^1^H NMR as the intensity of the protons alpha to selenium slowly decreased over the course of the experiment while the intensity of the terminal enone protons simultaneously increased (Fig. 3a). Using 1,4-dioxane as an internal standard, we were able to monitor the concentrations of both 1 and 2 throughout the course of the reaction, confirming that the consumption of 1 tracks nicely with the production of 2 (Fig. 3b). While the slow-release time is likely due, in part, to the reversible nature of the β-elimination, this, along with the clean formation of 2, unambiguously confirms not only the ability, but the mechanism by which 1 releases H_2_Se in buffer.

Additional qualitative evidence for selenide release from 1 was inferred from an observation that a noticeable red film was always present inside of our NMR tubes upon the completion of our kinetics experiments. In aqueous buffer, H_2_Se is known to rapidly oxidize to Se^0^,^19^ which is what we suspected this was. To confirm, the red film was isolated, treated with excess triphenylphosphine, and analyzed by ^31^P NMR (Fig. 3c). As predicted, the formation of triphenylphosphine selenide was cleanly observed, confirming the identity of the red residue as Se^0^.^34^

We also note that during the course of the reaction, we did observe a very small amount of diselenide formation, which is easily attributable to the propensity of the released selenol (from a single β-elimination) to oxidize in a nonreducing environment. This perhaps suggests that the first and second elimination steps are not necessarily coupled (as depicted in Fig. 2). Nevertheless, we confirmed that under the same reaction conditions, the ensuing diselenide also cleanly eliminates to form enone 2 while delivering a payload of H_2_Se_2_, which is known to quickly disproportionate to H_2_Se and Se^0^ (Fig. S2†).^35^

**Fig. 4:**
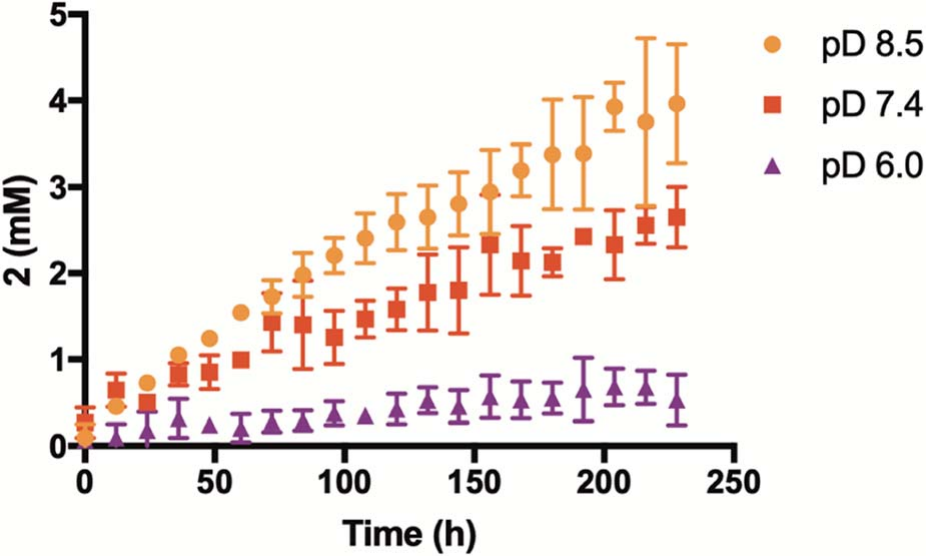
Effect of pD on enone (2) formation and selenide donation from 1 (2.5 mM). Reactions were run at ambient temperature and in a 1 : 1 mixture of CD_3_CN and deuterated phosphate buffer (50 mM). All reactions were performed in triplicate and plotted as the mean ± STDEV.

Having confirmed that the reaction mechanism proceeds through a β-elimination pathway, we suspected that the rate of H_2_Se release would be highly influenced by pH. To examine this, additional kinetic studies were run in deuterium buffer at a pD of 6 and 8.5 (Fig. 4). As anticipated, the formation of 2 was found to be greatly accelerated in a more basic environment. Conversely, its production was noticeably inhibited under slightly acidic conditions. These results suggest that γ-keto selenides are endowed with pH-control over their release of selenide, which may prove useful in certain applications.

After demonstrating the pH dependence on enone formation and selenide donation, we rationalized that the introduction of simple structural modifications that tuned α-proton acidity would provide a series of related donors with highly variable rates of H_2_Se liberation.

**Fig. 5:**
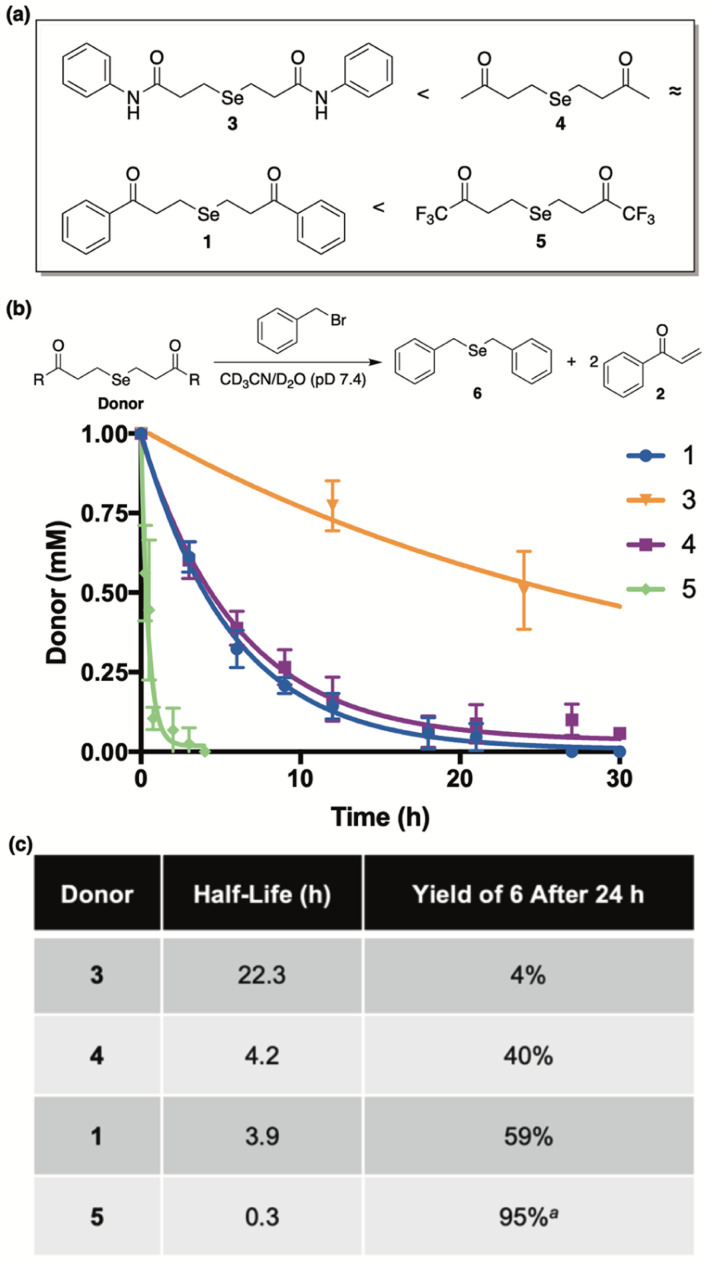
(a) Library of donors arranged in the order of increasing alpha proton acidity (5 being the most acidic). (b) Time-course for the decomposition of donors (1 mM) in the presence of benzyl bromide (4.4 mM). Reactions were run at ambient temperature and in a 1 : 1 mixture of CD_3_CN and deuterated phosphate buffer (50 mM, pD 7.4). 1,4-Dioxane was used as an internal standard to determine concentrations at various time points. Curves were fitted to obtain pseudo-first-order rate constants and half-lives (*t*_1/2_). All reactions were performed in triplicate and plotted as the mean ± STDEV. (c) Comparisons between donor half-life and the yield of 6 after a 24 hour incubation period. ^*a*^Combined yield of mono and diselenide derivatives of benzyl bromide after a 7 hour incubation period.

Our library of donors is depicted in Fig. 5a and is arranged in the order of increasing α-proton acidity (3 < 4 ≈ 1 < 5). For these experiments we employed benzyl bromide as an electrophilic trap to confirm selenide release and to render the reaction irreversible. Once again, a 1 : 1 mixture of CD_3_CN and deuterated phosphate buffer (50 mM, pD 7.4) was employed, and the reaction progress was monitored by ^1^H NMR using 1,4-dioxane as an internal standard.

Both donor consumption (Fig. 5b) and the formation of 6 were examined throughout the course of the reaction and the results are summarized in Fig. 5c. In general, alpha proton acidity was found correlate perfectly with both the rate of selenide release and the ensuing yield of 6. For example, 1 and 4 were found to have similar rates of decomposition (*t*_1/2_ = 3.9 h and 4.2 h, respectively), while giving rise to similar yields of 6 (59% and 40%, respectively). Being an amide with significantly decreased alpha proton acidity, donor 3 was found to be by far the most stable (*t*_1/2_ = 22.3 h), as expected, and its yield of 6 was exceptionally low, indicating very little selenide release. Conversely donor 5, being far and away the most acidic, was discovered to decompose almost instantly in buffer (*t*_1/2_ = 0.3 h), while engendering the highest yield of 6 in just a few hours. It is interesting to note that in the case of 5, both the mono and diselenide derivatives of benzyl bromide were observed by NMR (Fig. S3†), and its reported yield (95%) was calculated from the combined concentrations of both species. Contrarily, only the monoselenide derivative (6) was observed for reactions between donors 1, 3, and 4 and benzyl bromide.

To unambiguously confirm H_2_Se release from γ-keto selenides, we performed an additional trapping experiment where donor 1 was incubated in a tube separate from the electrophilic trap.^29^ For this experiment, we employed iodoacetamide as the electrophilic agent as the ensuing product from its trapping of volatilized H_2_Se would be easily discernible by high-resolution mass spectrometry. To accomplish this, the solution of 1 was acidified and sparged with argon to facilitate H_2_Se liberation into the headspace of the tube. There, its transfer through a carefully placed cannula needle would provide access to the separate, trapping solution. Using high-resolution mass spectrometry, the expected selenide product was clearly visible in the trapping solution, confirming the ability of 1 to directly release H_2_Se (Fig. S5†).

**Fig. 6:**
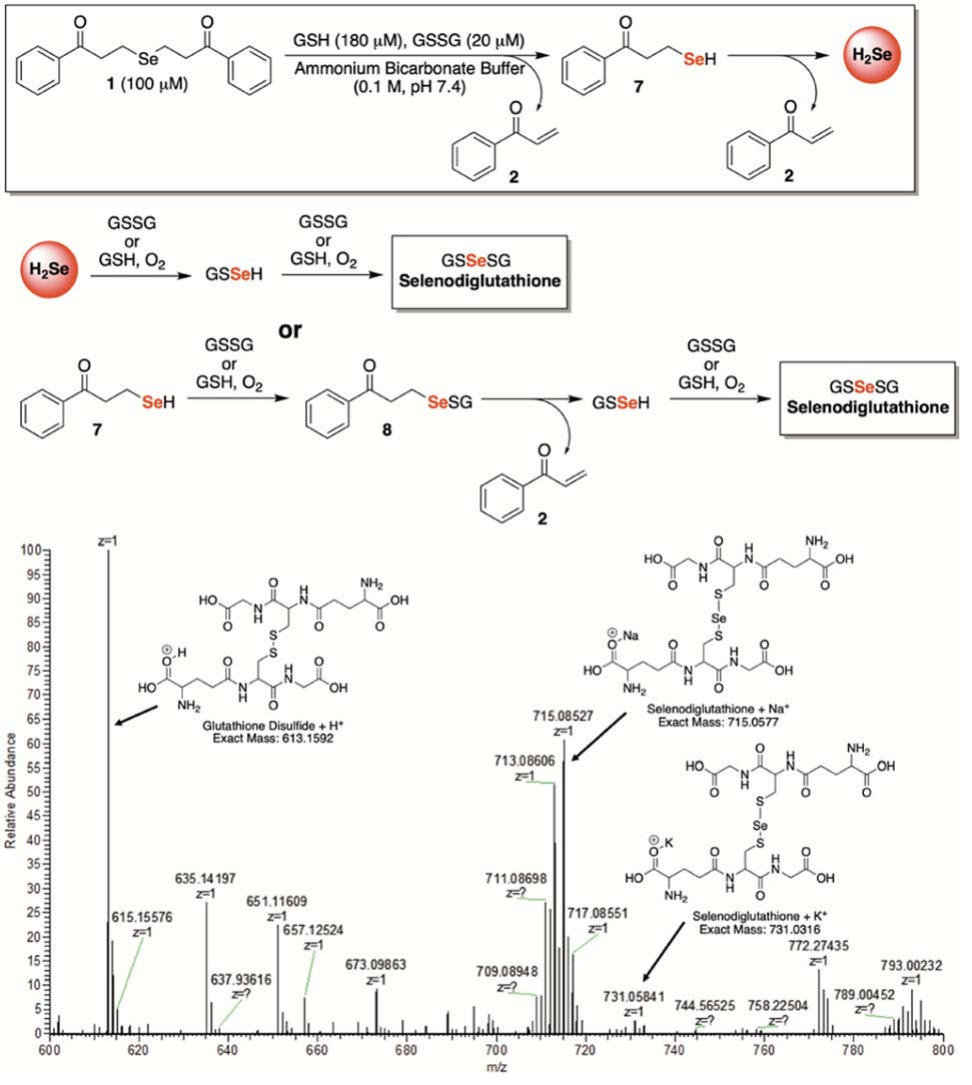
Confirmation of selenodiglutathione formation, a known H_2_Se precursor, upon exposure of 1 (100 μM) to a biologically relevant redox buffer consisting of glutathione (180 μM) and glutathione disulfide (20 μM) in ammonium bicarbonate buffer (0.1 M, pH 7.4).

In search of further evidence for H_2_Se release from γ-keto selenides, but under more biologically relevant conditions, we incubated 1 (100 μM) in a redox buffer consisting of glutathione (180 μM) and glutathione disulfide (20 μM) at room temperature and in an ammonium bicarbonate buffer (0.1 M, pH 7.4) (Fig. 6). In this setting, we surmised that both H_2_Se and/or 7—which would form from a single β-elimination—would have the potential to form glutathioneselenol (GSSeH), which could then react further under the reaction conditions to generate the more stable selenodiglutathione (GSSeSG) species that we might be able to observe directly by mass spectrometry. Indeed, when an aliquot of the reaction mixture was analyzed by direct infusion MS, the mass corresponding to GSSeSG (GSSeSG + Na^+^) was clearly visible and appeared to form in an appreciable amount, especially given its relative abundance to that of glutathione disulfide (GSSG + H^+^) (Fig. 6). This result unequivocally confirms the ability of γ-keto selenides, like 1, to produce H_2_Se under biological conditions, since it establishes their capacity to form biologically relevant reactive selenium species that are known to be in equilibrium with H_2_Se (Fig. 1).

Lastly, we aimed to evaluate this new series of donors within a cellular environment. The anticancer activity of H_2_Se has been recorded in earlier studies and appears to be quite promising. Depending on cellular conditions, H_2_Se can function as either a potent reductant or pro-oxidant, inducing reductive or oxidative stress, respectively, in cancer cells leading to apoptosis.^36^ Given that cancer cells exhibit a higher intracellular pH compared to normal cells,^37^ we speculated that γ-keto selenides, and their more efficient release of selenide under more basic conditions (Fig. 4), might be especially advantageous at inhibiting cancer cell growth.

**Fig. 7:**
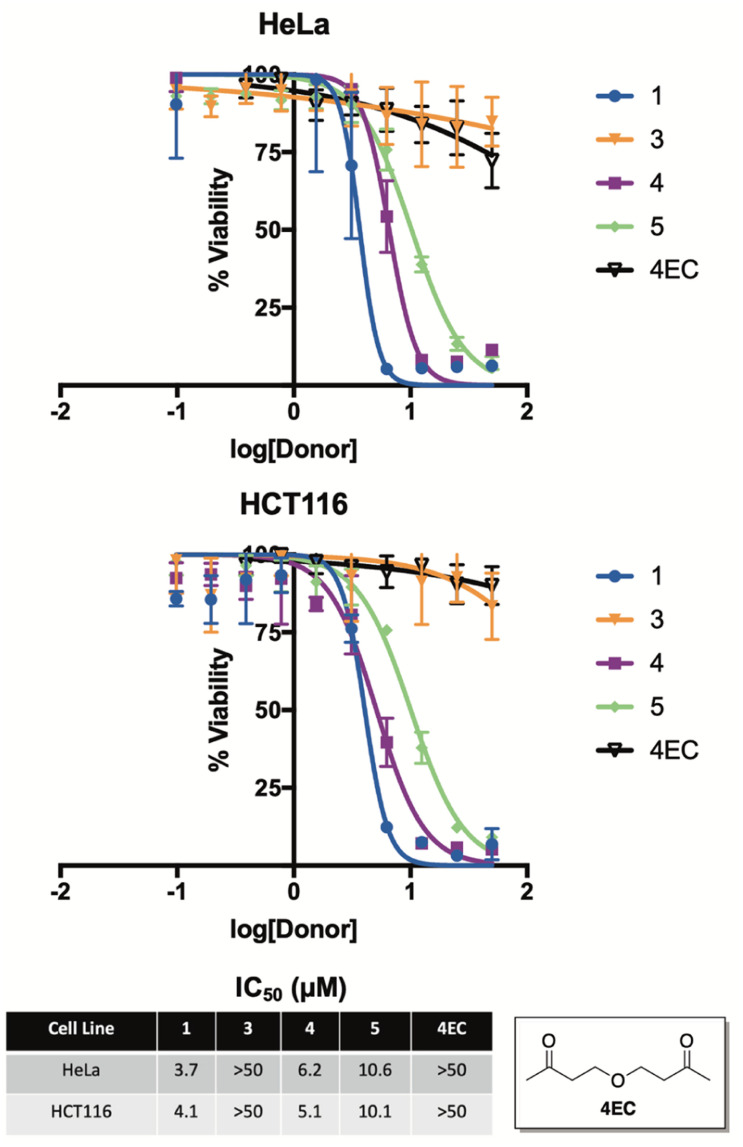
Cell growth inhibition of 1, 3, 4, 5, and 4EC (ether control) in HeLa (human cervical cancer) and HCT116 (human colon cancer) cells in culture. IC_50_ values were determined after a 24 hour incubation period and are reported as the average ± STDEV from three independent assays.

To examine this premise, we screened compounds 1, 3, 4, and 5 for growth inhibition activity against HeLa (human cervical cancer) and HCT116 (human colon cancer) cells in culture (Fig. 7). Not surprisingly, compound 3—which was found to be completely stable in buffer for months at a time and only liberated trace amounts of selenide, even while in the presence of benzyl bromide—was completely inactive in both cell lines. In stark contrast, compounds 1, 4, and 5 each displayed low micromolar activity against both HeLa and HCT116 cells, which is consistent with the anticancer activity of previously reported H_2_Se donors.^36,38^

As a key control, we also synthesized and assessed the anticancer activity of 4EC, an oxygen congener of 4, to help differentiate the cancer cell growth inhibitory of effects of H_2_Se from the other organic components of the reaction. As expected, 4EC displayed similar activity to that of 3 in both HeLa and HCT116 cells in culture, suggesting that it is, in fact, the released selenide from 1, 4, and 5 that is likely to be responsible for the promising anticancer activity of these compounds.

It is also interesting to note that 5, which releases H_2_Se at more than an order of magnitude faster than both 1 and 4 (Fig. 5), was about two-fold less potent. Similar to the anticancer activity of slow-releasing H_2_S donors,^39^ this result is intriguing and perhaps suggests that the slow and sustained decomposition of 1 and 4, which results in cells being exposed to continuous low levels of H_2_Se for a prolonged period of time, is more beneficial to cancer cell growth inhibition than is the instantaneous surge in selenide concentration that is likely afforded by 5.

## Conclusions

In closing, we report that γ-keto selenides offer a valuable and highly versatile platform for pH-dependent, H_2_Se donation. Detailed mechanistic studies were carried out by ^1^H NMR, which confirm the mechanism of release as an α-deprotonation/β-elimination sequence. As such, accelerated rates of selenide liberation were observed at higher pH and with donors that possess more acidic alpha protons. We further established H_2_Se donation under more biologically relevant conditions by verifying the formation of selenodiglutathione upon γ-keto selenide exposure to a glutathione/glutathione disulfide redox buffer. Impressive anticancer activity was also observed with this class of donors in both HeLa and HCT116 cells in culture. Overall, these results establish γ-keto selenide-based donors as a valuable addition to the investigative toolkit for probing the chemical biology and therapeutic value of H_2_Se and their introduction is likely to spark additional interest and research into this burgeoning field of study.

## Data availability

The data that support the findings of this study are available from the corresponding author upon reasonable request.

## Author contributions

RAH: synthesis and characterization of compounds, mechanistic studies, data acquisition and analysis, and manuscript preparation. MEC: assisted with synthesis and characterization of compounds and data acquisition. CZ: cytotoxicity studies and data analysis. CC: assisted with cytotoxicity studies and data analysis. JCL: project conceptualization and supervision, acquisition of funding, data analysis, and manuscript preparation and editing.

## Conflicts of interest

The authors declare no conflicts of interest.

## Supplementary Material

SC-013-D2SC03533B-s001
