# Supplementary material for: Enol-mediated delivery of H2Se from γ-keto selenides: mechanistic insight and evaluation
Source: Chem Sci. 2022 Oct 19;13(44):13094–9. doi: 10.1039/d2sc03533b (PMC9667953; doi:10.1039/d2sc03533b)
Supplement: SC-013-D2SC03533B-s001 [file SC-013-D2SC03533B-s001.pdf]

Electronic Supplementary Material (ESI) for Chemical Science.
This journal is © The Royal Society of Chemistry 2022

Enol-Mediated Delivery of H_2Se from γ-Keto Selenides: Mechanistic Insight and Evaluation

Rynne A. Hankins, Molly E. Carter, Changlei Zhu, Mary Chen, and John C. Lukesh, III*

Contents **Page Number**

Contents	Page Number
I. General	S2
II. Safety and Handling	S2
III. Synthesis	S2
IV. Kinetic Studies	S8
V. Trapping Experiments with Benzyl Bromide	S10
VI. Confirmation of Se^0 Formation with Triphenylphosphine	S13
VII. Formation of Selenodiglutathione	S14
VIII: Gas Trapping Experiments	S14
IX. Cell Culturing and Cytotoxicity Studies	S15
X. References	S16
XI. NMR Spectra	S17

I. General

Commercial reagents were used without further purification unless stated otherwise. All glassware was oven or flame-dried and reactions were performed under an N_2(g) atmosphere. Dichloromethane (DCM) and tetrahydrofuran (THF) were dried over a column of alumina. Flash chromatography was performed with columns of 40–63 Å silica from Silicycle (Québec City, Canada). Thin-layer chromatography (TLC) was performed on plates of EMD 250-μm silica 60-F254. Preparative thin-layer chromatography (PTLC) was performed on Whatman silica gel (500-μm) F254 glass plates. The term "concentrated under reduced pressure" refers to removal of solvents and other volatile materials using a rotary evaporator while maintaining the water-bath temperature below 40 °C. Residual solvent was removed from samples at high vacuum (<0.1 torr) using an Edwards RV5 pump. All NMR spectra were acquired at ambient temperature with a Bruker Ascend™ 400 MHz spectrometer and referenced to TMS or residual protic solvent. High resolution mass spectra were acquired using a Thermo Orbitrap LTQ XL (ESI). Adjustments in pH/pD for buffered solutions were accomplished with a Fisher Scientific Accumet AE150 pH meter. Cell viability was assessed spectrophotometrically using a BioTek SYNERGY-HTX multi-mode 96-well plate reader.

II. Safety and Handling

Hydrogen selenide is highly toxic and proper safety precautions must be in place when handling selenide salts and synthetic donors. All reactions and assays were performed in a well-ventilated fume hood and solutions containing donor we immediately quenched with silver nitrate prior to their disposal.

III. Synthesis

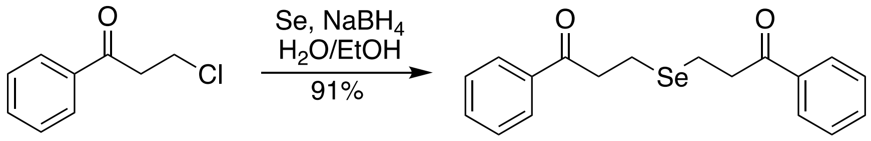

3-Chloropropiophenone **1**

To a solution of sodium borohydride (40 mg, 1.1 mmol) in a 1:1 solution of degassed ethanol and DI water (20 mL) was added selenium powder (39 mg, 0.5 mmol). The mixture was then heated to 50 °C under an atmosphere of nitrogen for 30 minutes, at which point the solution turned colorless and the selenium had completely dissolved. Next, 3-chloropropiophenone (168 mg, 1.0 mmol) was added and the reaction continued to stir at 50 °C until the starting material was observed to be consumed by TLC. The reaction was then cooled to room temperature and extracted with dichloromethane (50 mL). The organic layer was isolated, dried over anhydrous sodium sulfate, and concentrated under reduced pressure. The desired product was isolated by silica gel chromatography (gradient of 100% hexanes to 10% ethyl acetate in hexanes) to yield **1** as a white solid (158 mg, 91% yield).

^{1}H NMR (400 MHz, $CDCl_3$): δ = 8.00–7.93 (m, 4H), 7.61–7.54 (m, 2H), 7.51–7.43 (m, 4H), 3.43 (t, *J* = 7.2 Hz, 4H), 2.99 (t, *J* = 7.2 Hz, 4H); **^{13}C NMR (101 MHz, $CDCl_3$)** δ = 198.7, 136.6, 133.3, 128.7, 128.1, 39.9, 17.5; **^{77}Se NMR (76 MHz, $CDCl_3$)** δ = 200.0; **HRMS** calculated for [$C_{18}H_{19}O_2Se^+$] (M+H$^+$) requires *m/z* = 347.0544, found 347.0535.

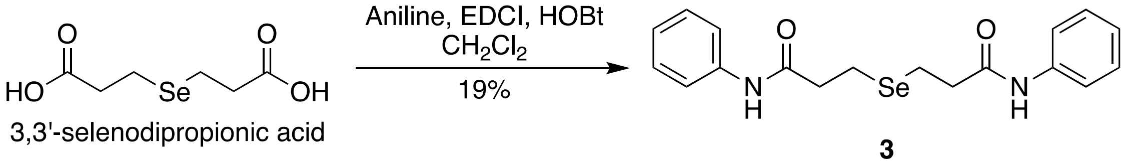

3

3,3'-selenodipropionic acid (320 mg, 1.4 mmol)[1], EDCI hydrochloride (600 mg, 3.1 mmol), HOBt (97 mg, 0.7 mmol), and aniline (286 µL, 3.1 mmol) were combined in dry dichloromethane (10 mL) and allowed to stir at ambient temperature under an N_2(g) atmosphere overnight. The mixture was further diluted with dichloromethane (100 mL) and washed with DI water (50 mL). The organic layer was collected, dried over anhydrous sodium sulfate, concentrated under reduced pressure, and purified by silica gel chromatography (gradient of 100% dichloromethane to 95:5 dichlormethane/methanol). The ensuing mixture was further triturated with dichloromethane (10 mL) to yield **3** as a white solid (100 mg, 19% yield).

^{1}H NMR (400 MHz, DMSO): δ = 9.96 (s, 2H), 7.63–7.55 (m, 4H), 7.35–7.25 (m, 4H), 7.09–6.99 (m, 2H), 2.84 (td, *J* = 7.1, 1.2 Hz, 4H), 2.74 (td, *J* = 7.0, 1.2 Hz, 4H); **^{13}C NMR (101 MHz, DMSO)** δ = 170.4, 139.6, 129.2, 123.6, 119.5, 38.1, 18.7; **^{77}Se NMR (76 MHz, DMSO)** δ = 186.3; **HRMS** calculated for [$C_{18}H_{21}N_2O_2Se^+$] (M+H$^+$) requires *m/z* = 377.0762, found 377.0764.

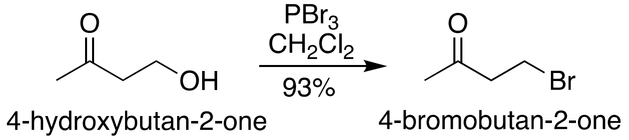

Phosphorous tribromide (475 µL, 5.0 mmol) was added dropwise to a cooled solution (using an ice bath) of 4-hydroxybutan-2-one (861 µL, 10.0 mmol) in dry dichloromethane (10 mL). After two hours, consumption of the starting material was inferred by TLC, and the mixture was directly concentrated under reduced pressure via rotary evaporation. The resultant oil was loaded onto a silica gel column and eluted (using a gradient of 100% hexanes to 25% ethyl acetate in hexanes) to afford 4-bromobutan-2-one as an oil (1.40 g, 93% yield).

^{1}H NMR (400 MHz, $CDCl_3$): δ =3.55 (t, J = 6.8 Hz, 2H), 3.04 (t, J = 6.7 Hz, 2H), 2.19 (s, 3H); **^{13}C NMR (101 MHz, $CDCl_3$)** δ = 205.3, 46.0, 30.2, 25.2.

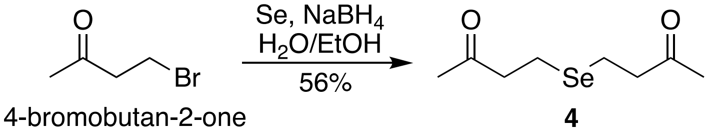

To a round bottom flask containing sodium borohydride (79 mg, 2.1 mmol) in a 1:1 solution of degassed ethanol and DI water (20 mL) was added selenium powder (78 mg, 1.0 mmol). The reaction mixture was heated to 50 °C under a nitrogen atmosphere for 30 minutes, at which point the solution turned colorless and the selenium had dissolved. Next, 4-bromobutan-2-one (300 mg, 2.0 mmol) was added, and the reaction was allowed to stir at 50 °C until the starting material was deemed to be consumed by TLC. The reaction was cooled to room temperature, diluted with DI water (20 mL) and extracted with dichloromethane (20 mL). The organic layer was dried over anhydrous sodium sulfate, concentrated under reduced pressure, and the resultant residue was purified by silica gel chromatography (gradient of 100% hexanes to 25% ethyl acetate in hexanes) to yield **4** as an oil (123 mg, 56% yield).

^{1}H NMR (400 MHz, $CDCl_3$): δ = 2.93–2.81 (m, 4H), 2.81–2.69 (m, 4H), 2.17 (s, 6H); **^{13}C NMR (101 MHz, $CDCl_3$)** δ = 207.1, 44.5, 30.1, 16.8; **^{77}Se NMR (76 MHz, $CDCl_3$)** δ = 197.4; **HRMS** calculated for [$C_8H_{15}O_2Se^+$] (M+H$^+$) requires *m/z* = 223.0231, found 223.0233.

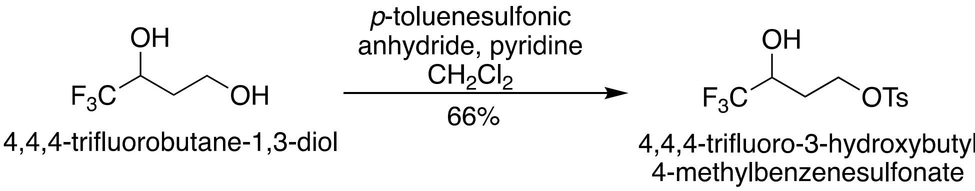

To a solution of 4,4,4-trifluorobutane-1,3-diol[2] (641 mg, 4.4 mmol) in dry dichloromethane (20 mL) was added *p*-toluenesulfonic anhydride—freshly recrystallized from hot hexanes—(1.83 g, 5.6 mmol),[3] and pyridine (1.8 mL, 22.3 mmol), and the resultant mixture was allowed to stir at room temperature overnight. Upon confirming the consumption of starting material by TLC, the crude mixture was concentrated under reduced pressure and purified by silica gel chromatography (slow gradient of 100% hexanes to 25% ethyl acetate in hexanes) to afford 4,4,4-trifluoro-3-hydroxybutyl 4-methylbenzenesulfonate as a yellow oil (1.03 g, 79%).

^{1}H NMR (400 MHz, $CDCl_3$): δ = 7.85 – 7.77 (m, 2H), 7.42 – 7.33 (m, 2H), 4.33 (td, *J* = 10.4, 3.7 Hz, 1H), 4.20 – 4.13 (m, 2H), 2.59 (d, *J* = 23.3 Hz, 1H), 2.46 (s, 3H), 2.15 – 2.05 (m, 1H), 1.86 (ddt, *J* = 14.6, 10.7, 3.8 Hz, 1H). **^{13}C NMR (101 MHz, $CDCl_3$)** δ = 145.3, 132.6, 130.0, 127.9, 126.2 (q, *J* = 190.6), 66.7 (q, *J* = 31.86), 65.5, 29.1, 21.7; **^{19}F NMR (376 MHz, $CDCl_3$)**: δ = −80.1 (d, *J* = 6.6 Hz); **HRMS** calculated for [$C_{11}H_{14}F_3O_4S^+$] (M+H$^+$) requires *m/z* = 299.0559, found 299.0554.

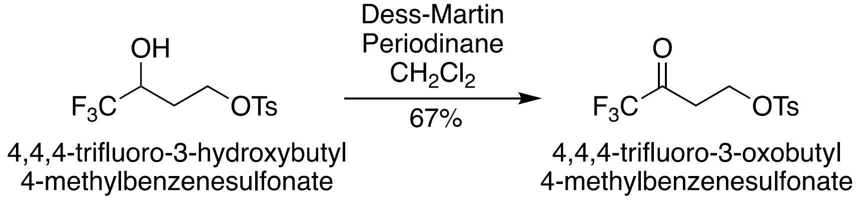

To a solution of 4,4,4-trifluoro-3-hydroxybutyl 4-methylbenzenesulfonate (260 mg, 0.9 mmol) in dry dichloromethane (10 mL) was added Dess-Martin periodinane (559 mg, 1.3 mmol). After stirring overnight, the reaction was concentrated under reduced pressure, and purified by silica gel chromatography (gradient of 100% hexanes to 50% ethyl acetate in hexanes) to afford an oily mixture of both ketone and hydrate. Further evaporation under high vacuum provided the ketone (4,4,4-trifluoro-3-oxobutyl 4-methylbenzenesulfonate) as a white solid (178 mg, 67% yield).

^{1}H NMR (400 MHz, $CDCl_3$): δ = 7.86–7.75 (m, 2H), 7.41–7.32 (m, 2H), 4.34 (t, *J* = 6.0 Hz, 2H), 3.13 (t, *J* = 6.0 Hz, 2H), 2.47 (s, 3H); **^{13}C NMR (101 MHz, $CDCl_3$)** δ = 187.6 (q, *J* = 37.0 Hz), 145.4, 132.3, 130.0, 128.1, 115.1 (q, *J* = 291.2 Hz), 62.4, 35.9, 21.7; **^{19}F NMR (376 MHz, $CDCl_3$)**: δ = −79.5; **HRMS** calculated for [$C_{11}H_{12}F_3O_4S^+$] ($M+H^+$) requires *m/z* =297.0402, found 297.0402.

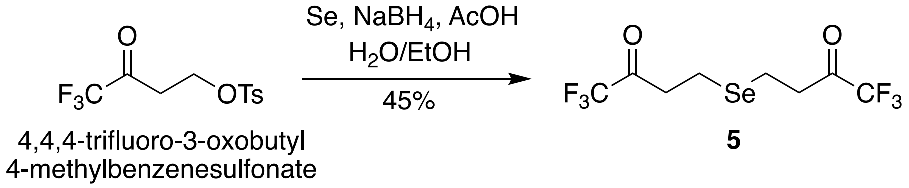

5

To a round bottom flask containing sodium borohydride (11 mg, 0.3 mmol) in a 1:1 solution of degassed ethanol and DI water (10 mL) was added selenium powder (9 mg, 0.1 mmol). Once the vigorous bubbling had subsided, the mixture was heated to 50 °C under nitrogen until the reaction turned colorless and the selenium had completely dissolved. At this point, the reaction was cooled to room temperature using a water bath and acetic acid (200 μL) and 4,4,4-trifluoro-3-oxobutyl 4-methylbenzenesulfonate (78 mg, 0.26 mmol in degassed ethanol (3 mL) were both quickly added. After stirring for an additional hour, the mixture was extracted with dichloromethane (20 mL) and the organic layer was dried over anhydrous sodium sulfate and concentrated under reduced pressure. The crude product was quickly purified via a silica gel plug (50% ethyl acetate in hexanes) and, after further evaporation under high vacuum, afforded **5** (15 mg, 45% yield). Analytically pure samples of **5** for kinetics and cell testing were obtained by preparative thin layer chromatography (1:3 hexanes:ethyl acetate) immediately prior to use.

^{1}H NMR (400 MHz, CDCl$_3$): δ = 3.21–3.16 (m, 4H), 2.87 (t, *J* = 6.9 Hz, 4H); **^{13}C NMR (101 MHz, CDCl$_3$)** δ = 190.3 (q, *J* = 35.9 Hz), 113.9 (q, *J* = 291.7 Hz), 38.0, 15.3; **^{19}F NMR (376 MHz, CDCl$_3$)**: δ = −79.3; **^{77}Se NMR (76 MHz, CDCl$_3$)** δ = 218.2; **HRMS** calculated for [$C_{10}H_{17}F_6O_4Se^+$] (M+H$^+$ of the methanol *bis*-hemihydrate) requires *m/z* = 393.0199, found 393.0042.

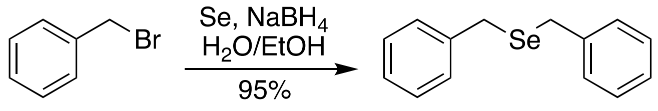

benzyl bromide **6**

To a solution of sodium borohydride (76 mg, 2.0 mmol) in degassed DI water (5 mL) and ethanol (5 mL) was added selenium powder (80 mg, 1.0 mmol). The mixture was then heated to 50 °C under nitrogen for 30 minutes, at which point the solution turned colorless and the selenium had completely dissolved. Next, neat benzyl bromide (283 µL, 2.4 mmol) was introduced and the reaction continued to stir at 50 °C until the starting material was observed to be consumed by TLC. The reaction was then cooled to room temperature and extracted with dichloromethane (30 mL). The organic layer was collected, dried over anhydrous sodium sulfate, and concentrated under reduced pressure. The product was isolated by silica gel chromatography (gradient of 100% hexanes to 10% ethyl acetate in hexanes) to yield **6** as a yellow oil (247 mg, 95% yield).

^{1}H NMR (400 MHz, CDCl$_3$): δ = 7.36–7.15 (m, 10H), 3.72 (s, 4H); **^{13}C NMR (101 MHz, CDCl$_3$)** δ = 139.2, 129.0, 128.5, 126.7, 27.6; **^{77}Se NMR (76 MHz, CDCl$_3$)** δ = 333.4; **HRMS** calculated for [$C_{14}H_{15}Se^+$] (M+H$^+$) requires *m/z* = 263.0333, found 263.0333.

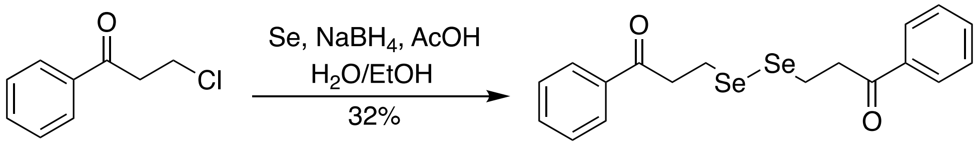

3-Chloropropiophenone **9**

To a solution of sodium borohydride (40 mg, 1.1 mmol) in a 1:1 solution of degassed ethanol and DI water (20 mL) was added selenium powder (40 mg, 0.5 mmol). The mixture was then heated to 50 °C under nitrogen for 30 minutes, at which point the solution turned colorless and the selenium had completely dissolved. A second portion of selenium powder (40 mg, 0.5 mmol) was then delivered, and the mixture was allowed to react at 50 °C for an additional 30 minutes. At this point, the reaction was cooled to room temperature using a water bath and acetic acid (60 µL) and 3-chloropropiophenone (168 mg, 1.0 mmol) in degassed THF (5 mL) were both quickly added. The reaction was then left to stir at room temperature until the starting material was deemed to have been consumed by TLC. The reaction was then diluted with DI water (20 mL) and extracted with ethyl acetate (20 mL). The organic layer was isolated, dried over anhydrous sodium sulfate, and concentrated under reduced pressure. The resultant crude product was

purified by silica gel chromatography (gradient of 100% hexanes to 10% ethyl acetate in hexanes) to yield **9** as a yellow solid (67 mg, 32% yield).

^{1}H NMR (400 MHz, $CDCl_3$): δ = 7.93–7.87 (m, 4H), 7.54–7.47 (m, 2H), 7.44–7.36 (m, 4H), 3.44 (t, *J* = 7.0 Hz, 4H), 3.19 (t, *J* = 7.0 Hz, 4H); **^{13}C NMR (101 MHz, $CDCl_3$)** δ = 198.5, 136.5, 133.4, 128.7, 128.1, 40.2, 22.8; **^{77}Se NMR (76 MHz, $CDCl_3$)** δ = 329.0; **HRMS** calculated for [$C_{18}H_{19}O_2Se_2^+$] (M+H$^+$) requires *m/z* 426.9711 =, found 426.9706.

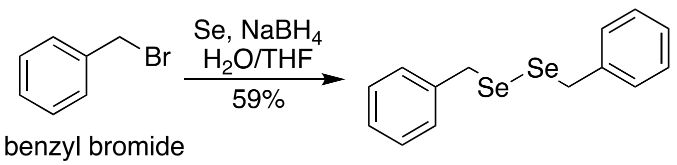

10

To a solution of sodium borohydride (79 mg, 2.1 mmol) in degassed DI water (5 mL) was added a portion selenium powder (83 mg, 1.05 mmol). The mixture was then heated to 50 °C under nitrogen for 30 minutes, at which point the solution turned colorless and the selenium had completely dissolved. A second portion of selenium powder (83 mg, 1.05 mmol) was then delivered, and the mixture was allowed to react at 50 °C for an additional 30 minutes. Benzyl bromide (238 µL, 2.0 mmol) in THF (10 mL) was then added and the reaction was allowed to stir overnight at 50 °C. Upon cooling to room temperature, the reaction mixture was diluted with dichloromethane (20 mL) and washed with DI water (20 mL). The organic layer was isolated, dried over anhydrous sodium sulfate, and concentrated under the reduced pressure. The resultant oil was purified by silica gel chromatography (100% hexanes) to afford **10** as a yellow oil (201 mg, 59% yield).

^{1}H NMR (400 MHz, $CDCl_3$): δ = 7.34 – 7.18 (m, 10H), 3.83 (s, 4H); **^{13}C NMR (101 MHz, $CDCl_3$)** δ = 139.0, 129.0, 128.5, 127.1, 32.6; **^{77}Se NMR (76 MHz, $CDCl_3$)** δ = 402.7; **HRMS** calculated for [$C_{14}H_{18}NSe_2^+$] (M+NH$_4^+$) requires *m/z* =359.9764, found 359.9769 (infused with Ammonium bicarbonate)

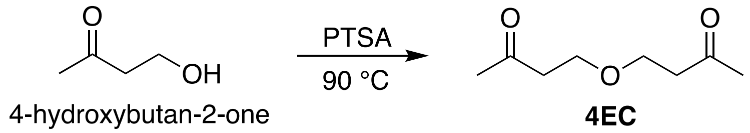

4EC

4-hydroxybutan-2-one (861 µL, 10.0 mmol) was added neat to a round bottom flask with *p*-toluenesulfonic acid monohydrate (48 mg, 0.25 mmol) and heated to 90 °C under argon overnight. The mixture was cooled to room temperature, diluted with diethyl ether (40 mL) and washed with deionized water (40 mL). The organic layer was isolated, dried over anhydrous sodium sulfate, and concentrated under the reduced pressure. The resultant oil was purified by silica gel chromatography (gradient of 100% hexanes to 50% diethyl ether in hexanes) to yield **4EC** as an oil (169 mg, 21% yield).

^{1}H NMR (400 MHz, $CDCl_3$): δ = 3.69 (t, *J* = 6.2 Hz, 4H), 2.66 (t, *J* = 6.2 Hz, 4H), 2.17 (s, 6H); **^{13}C NMR (101 MHz, $CDCl_3$)** δ = 207.2, 65.9, 43.6, 30.4; **HRMS** calculated for [$C_8H_{14}NaO_3^+$] (M+Na^+) requires *m/z* =181.0841, found 181.0840.

IV. Kinetics Studies

Prior to the start of the experiment, deuterated buffer was prepared from a 50 mM solution of Na_2HPO_4 in D_2O and the respective pD values (8.5, 7.4, 6) were achieved with the addition of deuterium chloride (DCl). A standard solution of 1,4-dioxane was prepared at 31.25 mM in D_2O. Donor stock solution (**1** or **9**) was prepared at 75 mM in deuterated acetonitrile (acetonitrile-d3). For each experiment, acetonitrile-d3 (350 µL) was added to a conical tube followed by the dioxane standard solution (15 µL) and donor stock solution (25 µL). Deuterated phosphate buffer (50 mM, 360 µL) was then added to bring the final concentration of **1** (or **9**) to 2.5 mM and the dioxane standard to 0.625 mM. At this point, the solution was immediately transferred to an NMR tube and the cap was fastened with a small strip of parafilm to prevent evaporation. Experiments were run at ambient temperature and for 64 scans at each respective timepoint. To monitor reaction progress, the integration value for 1,4-dioxane (3.75 ppm) was normalized to 1 proton in each spectrum.

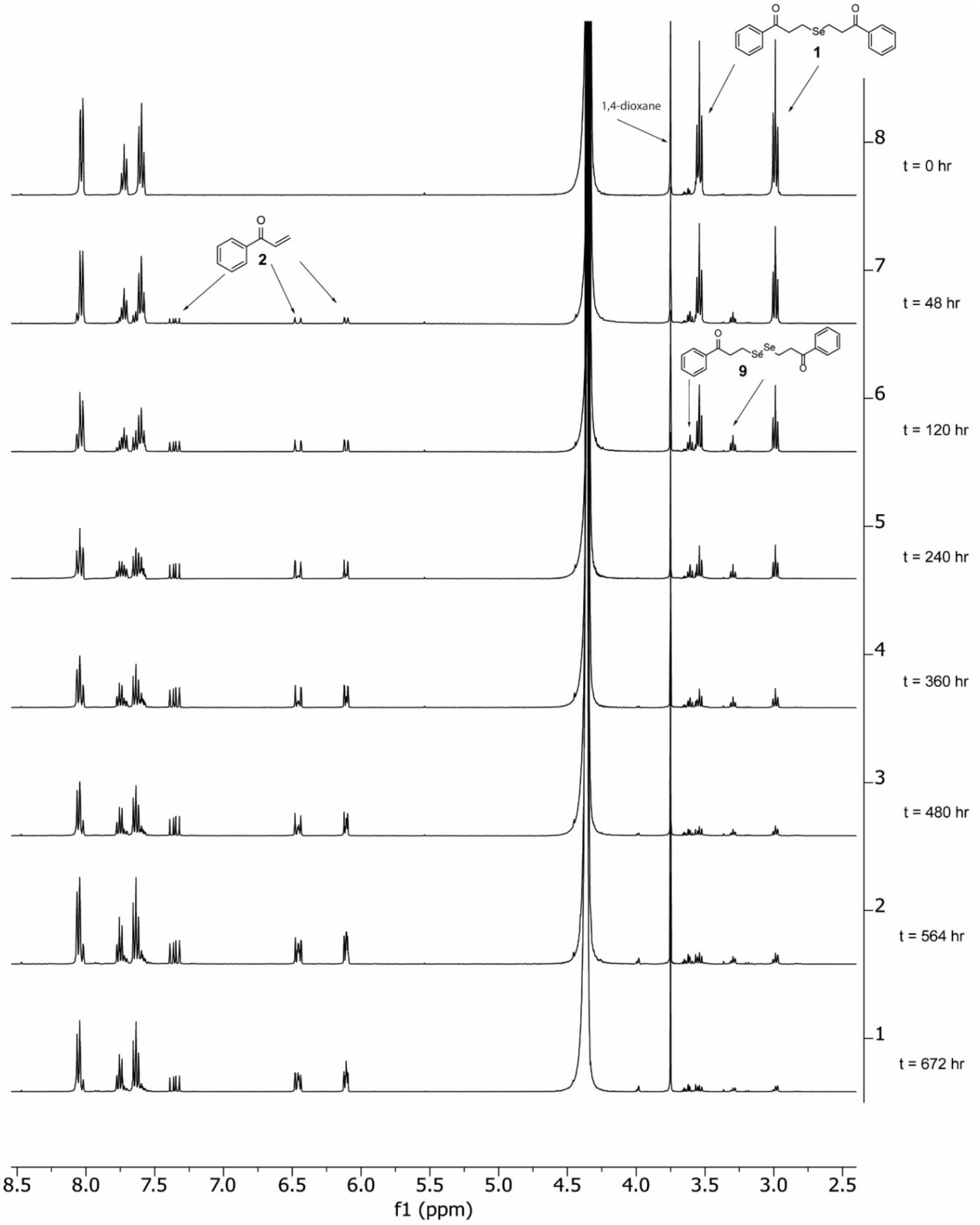

Figure S1. Time-course for the conversion of ketone **1** (2.5 mM) into enone **2** at ambient temperature and in a 1:1 mixture of CD_3CN and deuterated phosphate buffer (50 mM, pD 7.4). Reaction progress was monitored by ^1H NMR using 1,4-dioxane as an internal standard.

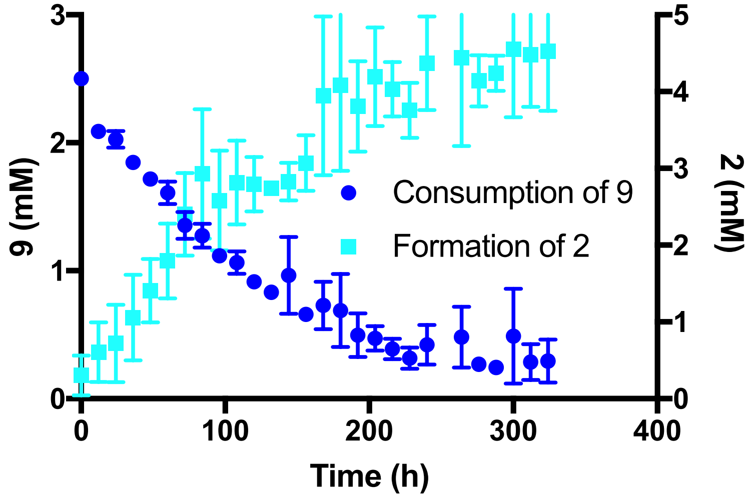

Figure S2. Time-course for the conversion of ketone **9** (2.5 mM) to enone **2** at ambient temperature and in a 1:1 mixture of CD_3CN and deuterated phosphate buffer (50 mM, pD 7.4). Reaction progress was monitored by ^1H NMR with 1,4 -dioxane being used as an internal standard to determine the concentration of **9** and **2** at different timepoints.

V. Trapping Experiments with Benzyl Bromide

Acetonitrile-d3 (321 μL) was added to a conical tube followed by a standard solution of 1,4-dioxane (31.25 mM) in D_2O (15 μL). Next, a stock solution of donor (**1**, **3**, **4**, or **5**, 75 mM, 10 μL) in acetonitrile-d3 was added. Immediately prior to the start of the experiment, of a 75 mM stock solution of benzyl bromide in acetonitrile-d3 (44 μL, 75 mM) was introduced followed by deuterated phosphate buffer (360 μL, 50 mM, pD of 7.4), bringing the final concentration of donor (**1**, **3**, **4**, or **5**) to 1 mM, benzyl bromide to 4.4 mM, and 1,4-dioxane to 0.625 mM. The solution was immediately transferred to an NMR tube and the cap was fastened with a small strip of parafilm to prevent evaporation. Experiments were run at ambient temperature and for 64 scans at each respective timepoint. To monitor reaction progress, the integration value for 1,4-dioxane (3.75 ppm) was normalized to 1 proton in each spectrum.

To confirm the identity of trapped selenium products, standards of benzyl monoselenide (**6**) and benzyl diselenide (**10**) were prepared (as described above) and their ^1H NMRs in a 1:1 mixture of acetonitrile-d3 and deuterated phosphate buffer (50 mM, pD 7.4) were referenced to 1,4-dioxane (3.75 ppm) (Figure S3a).

(a)

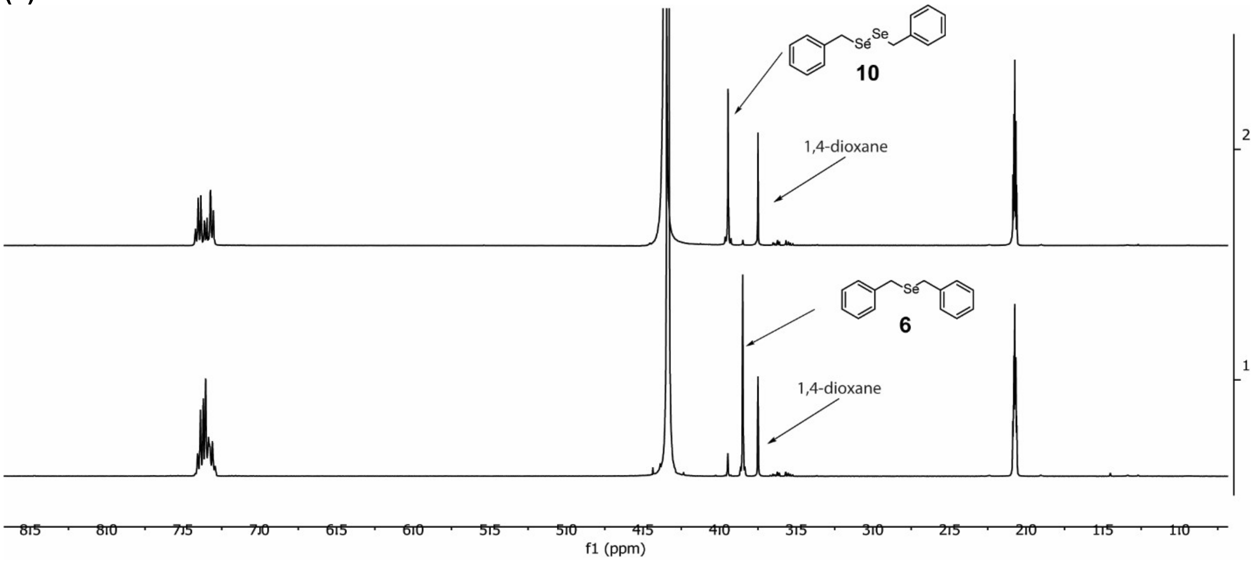
Se
Se
10
1,4-dioxane
Se
6
1,4-dioxane
2
1
8.5
8.0
7.5
7.0
6.5
6.0
5.5
5.0
4.5
4.0
3.5
3.0
2.5
2.0
1.5
1.0
f1 (ppm)

(b)

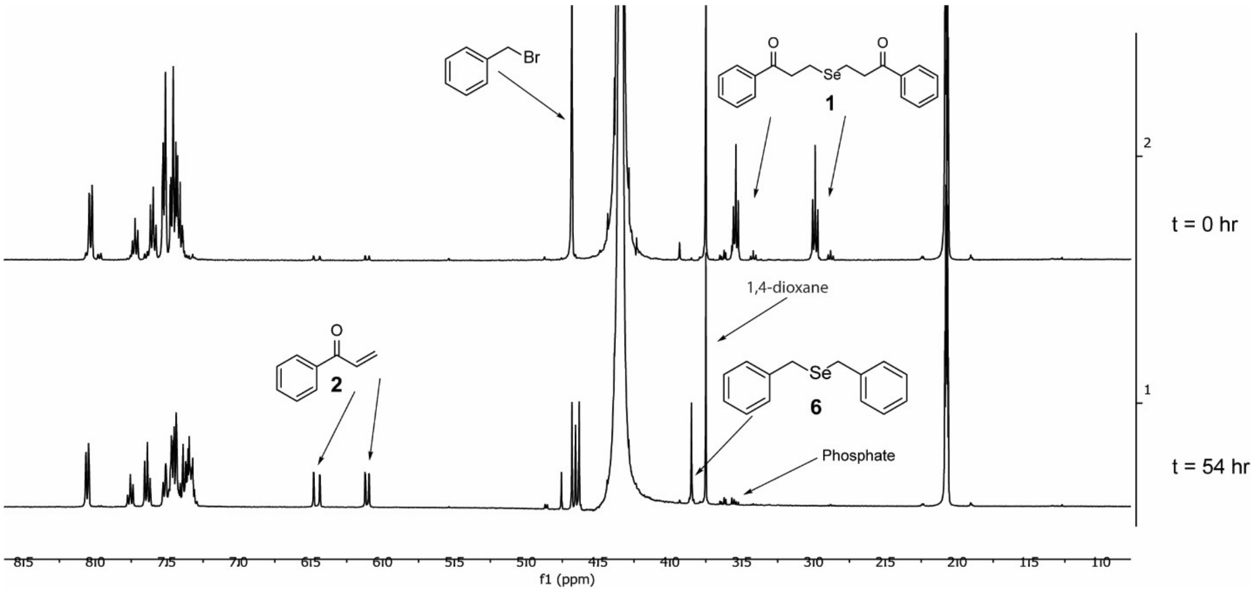
Br
O
O
Se
1
O
2
1,4-dioxane
Se
6
Phosphate
2
1
t = 0 hr
t = 54 hr
8.5
8.0
7.5
7.0
6.5
6.0
5.5
5.0
4.5
4.0
3.5
3.0
2.5
2.0
1.5
1.0
f1 (ppm)

(c)

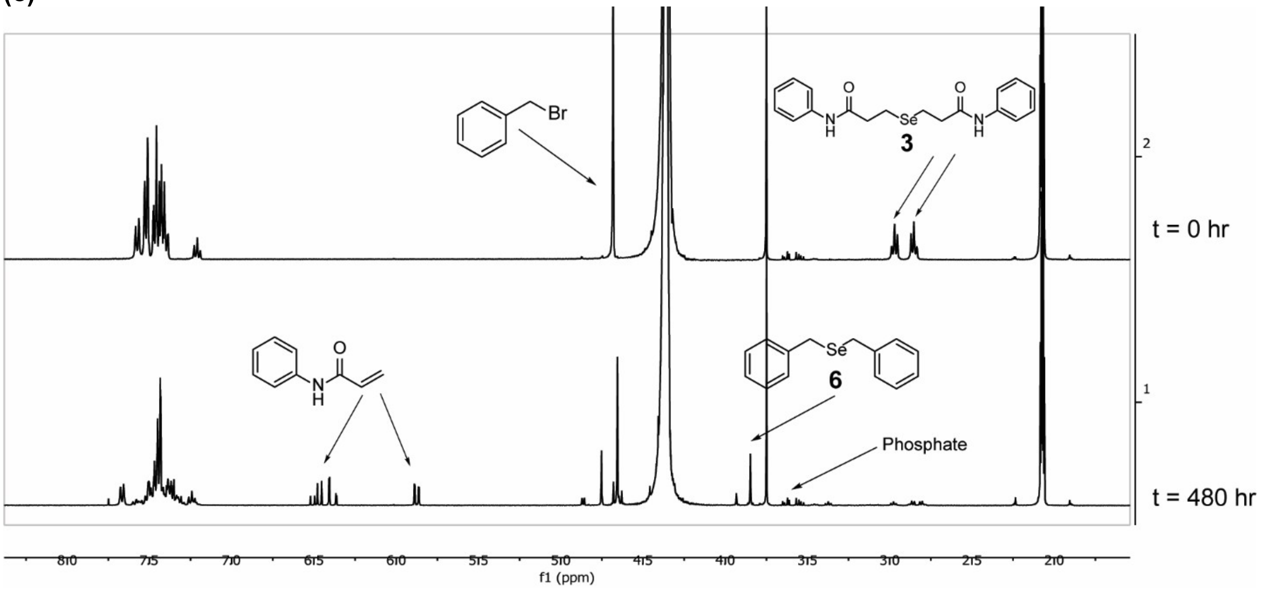

Br
Se
3
Se
6
Phosphate
t = 0 hr
t = 480 hr
f1 (ppm)

(d)

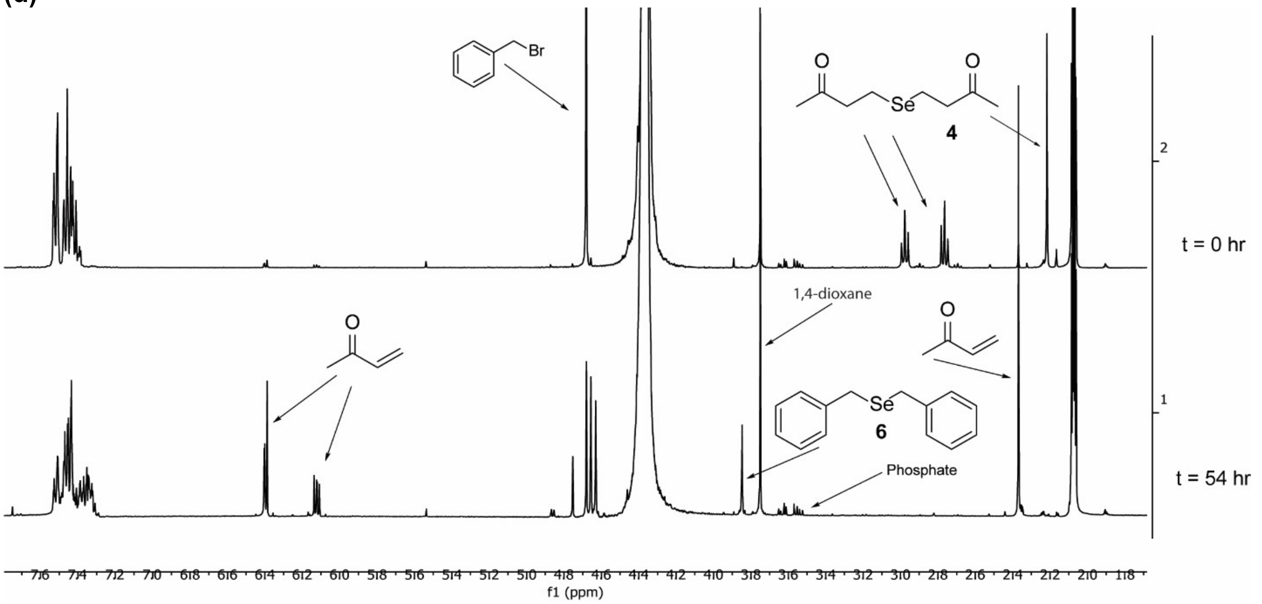

Br
Se
4
1,4-dioxane
Se
6
Phosphate
t = 0 hr
t = 54 hr
f1 (ppm)

(e)

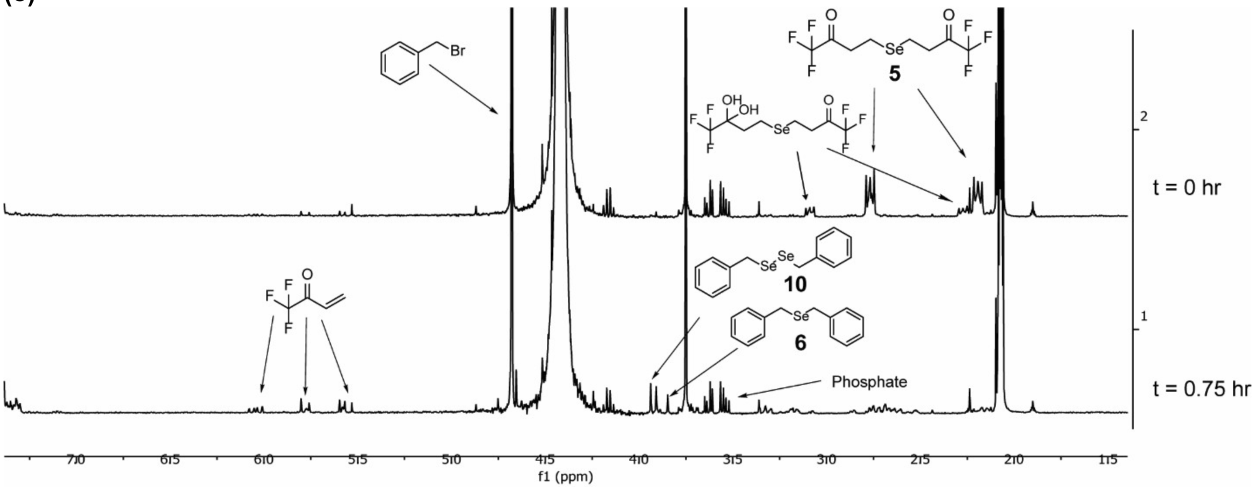

Figure S3. (a) Representative ^{1}H NMR spectra of **10** and **6** in a 1:1 mixture of CD_3CN and deuterated phosphate buffer (50 mM, pD 7.4), referenced to 1,4-dioxane (3.75 ppm). (b–e) Representative ^{1}H NMR spectra of donors **1** (b)**, 3** (c)**, 4** (d)**,** and **5** (e) (1.0 mM) treated with benzyl bromide (4.4 mM) resulting in the formation of monoselenide **6** (for donors **1**, **3**, **4**, **5**) and diselenide **10** (donor **5**). Reactions were run at ambient temperature in a 1:1 mixture of CD_3CN and deuterated phosphate buffer (50 mM, pD 7.4). 1,4-dioxane (3.75 ppm) was used as an internal standard to determine the concentrations compounds at different timepoints.

VI. Confirmation of Se^0 Formation with Triphenylphosphine

When kinetic experiments were deemed complete by ^{1}H NMR, solvent was decanted from the elemental selenium (Se^0), and the NMR tube was placed in an oven at 115 °C for approximately 30 minutes. After cooling to room temperature, a solution of triphenylphosphine in deuterated chloroform (700 µL, 5 mM) was then added to the NMR tube and allowed to react for 12 hours prior to analysis by ^{31}P NMR (Figure 3c).

To confirm the above results, 10 mg of elemental selenium (Alfa Aesar, 325 mesh, 99.5%) was added to an NMR tube and 700 µL of triphenylphosphine solution (5 mM) in deuterated chloroform was added. The reaction was allowed to progress for 6 hours prior to analysis by ^{31}P NMR (Figure S4).

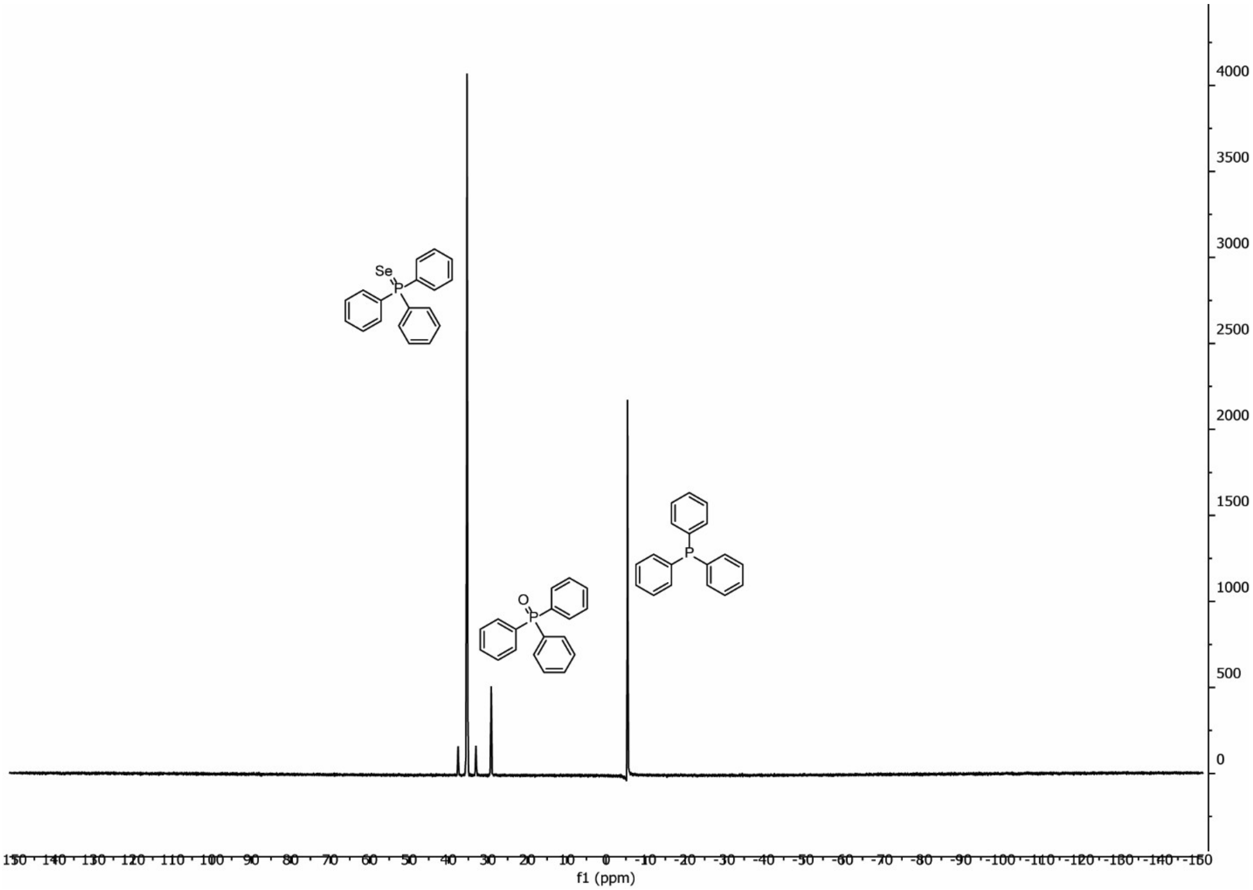

Figure S4. Resultant ^{31}P NMR spectrum from the test reaction between elemental selenium (Se^0) with triphenylphosphine. Triphenylphosphine selenide: 35.28 ppm, triphenylphosphine oxide: 29.17 ppm, triphenylphosphine: −5.41 ppm.

VII. Formation of Selenodiglutathione

To a 0.1 M solution of ammonium bicarbonate buffer (pH 7.4, 1455 µL) was added donor **1** (15 µL of a 10 mM solution in ethanol), glutathione (27 µL of a 10 mM solution in DI water) and glutathione disulfide (3 µL of a 10 mM solution in DI water) to give a final concentration of 100 µM **1**, 180 µM glutathione, and 20 µM glutathione disulfide. The reaction was warmed to 37 °C and, after reacting for 30 min, a 250 µL aliquot was removed and diluted with 750 µL of methanol with 0.1% formic acid. The resultant solution was analyzed via direct infusion mass spectrometry to observe the selenodiglutathione sodium adduct (Figure 6).

VIII: H_2Se Gas Trapping Experiments

Adopted procedure from Newton et al.[4] Donor **1** (1.0 g) was placed in a 15 mL Falcon® tube and dissolved in a mixture of THF (1 mL), deionized water (1 mL), and 4M HCl in 1,4-dioxane (10 mL). In a second tube, iodoacetamide (186 mg, 1 mmol) was dissolved in 10 mL of ammonium bicarbonate buffer (1.0 M, pH 8.5). Both tubes were then capped with septa and wrapped with parafilm. A vent needle was inserted into the septa

containing the trapping solution and two 4” cannula needles were used to connect the two tubes, ensuring that the tips of both needles remained in the headspace at all times. The first tube containing donor **1** was then pressurized with argon using a disposable needle, and the reaction was heated to 37 °C for three days. Aliquots of the trapping solution were monitored by mass spectrometry to confirm formation of trapped hydrogen selenide with iodoacetamide.

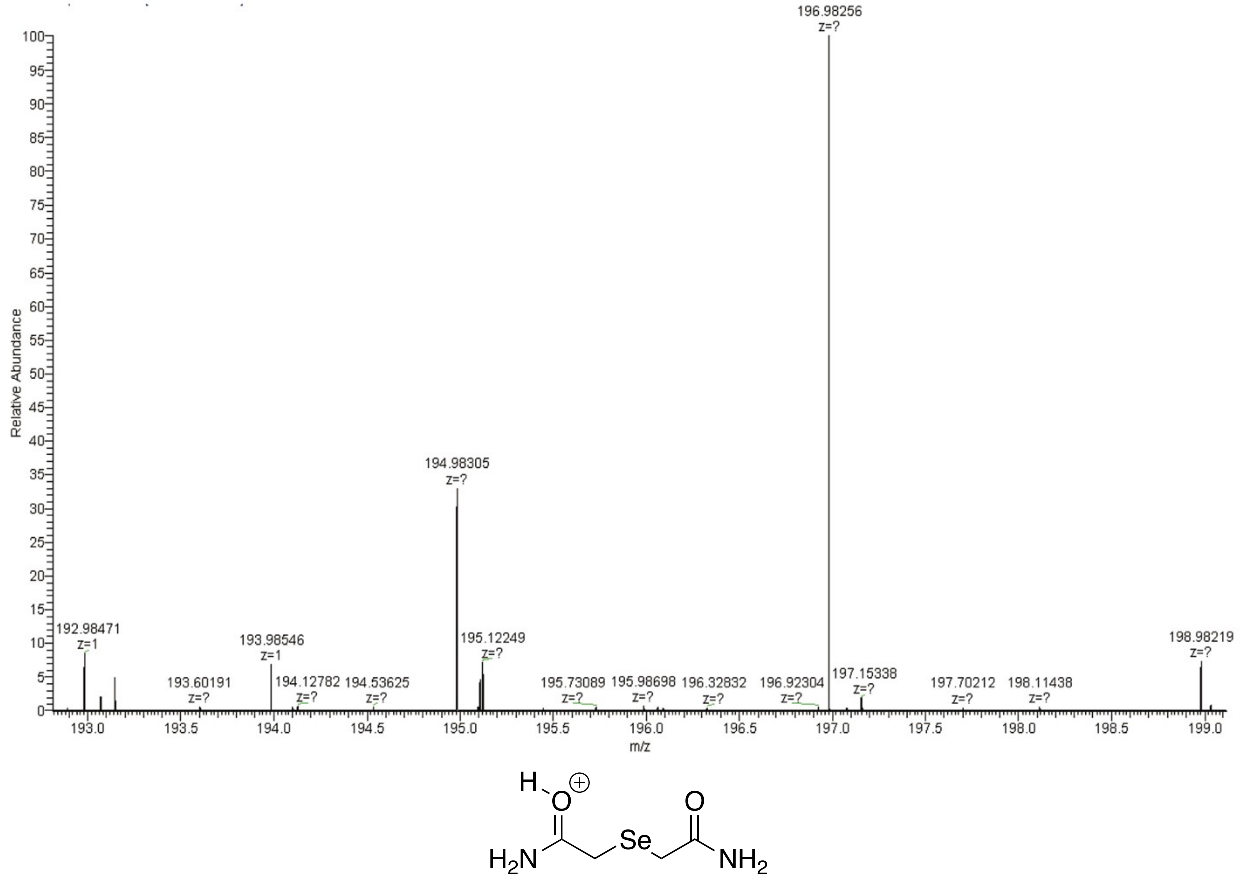

Chemical Formula: $C_4H_9N_2O_2Se^+$
Exact Mass: 196.9824
m/z: 196.9824 (100.0%), 194.9832 (47.9%), 192.9851 (18.9%), 198.9826 (17.6%), 193.9858 (15.4%), 197.9858 (4.3%), 195.9866 (2.1%), 190.9884 (1.8%)

Figure S5. High-resolution mass spectrum of volatilized hydrogen selenide gas trapped with iodoacetamide.

IX. Cell Culturing and Cytotoxicity Studies

Cell culturing

HeLa and HCT116 cells were cultured in Dulbecco’s Modified Eagle Medium (DMEM, Gibco) supplemented with 10% fetal bovine serum (FBS, Gibco), 1% L-glutamine, and 1% penicillin-streptomycin. Cells were incubated at 37 °C in an atmosphere of 5% CO_2 and 95% humidified air and split at 70–80% confluency every 2–3 days.

Cytotoxicity Studies

H_2Se donors (**1**, **3**, **4**, and **5**) and control (**4EC**) were tested for cell growth inhibition in HeLa (human cervical cancer) and HCT116 (human colorectal cancer) cells in culture. A population of cells (>1 x 10^6 cells/mL as determined with a hemocytometer) was diluted in DMEM, containing 10% fetal bovine serum, to give a final concentration of 50,000 cells/mL. To each well of a 96-well plate (Corning Costar) was added 100 μL of cell media solution to afford 5,000 cells/well. The plates were then incubated at 37 °C under an atmosphere of 5% CO_2 and 95% humidified air for 24 h. Fresh culture media (100 μL) containing 10% FBS was then added to each well (resulting in 200 μL per well) followed by the addition of test compounds (**1**, **3**, **4**, **5, or 4EC**) at various concentrations (delivered from an initial 40 mM stock solution in biological grade DMSO resulting in no more than 0.5% DMSO per well). Following the addition of donor compounds, cultures were incubated for an additional 24 h. A phosphatase assay was used to establish IC_{50} values as follows: The media was removed from each well and treated with 100 μL of phosphatase solution (100 mg of phosphatase substrate in 30 mL of 0.1 M NaOAc, pH 5.5, 0.1% Triton X-100 buffer). After an incubation period of 30 min, 50 μL of an NaOH solution (0.1 N) was added to each well and the absorbance at 405 nm was recorded. The percent cell viability at each donor concentration was calculated as follows:

$$\text{\% Viable Cells} = \frac{(\text{abs}_{\text{sample}} - \text{abs}_{\text{blank}})}{(\text{abs}_{\text{control}} - \text{abs}_{\text{blank}})} \times 100$$

X. References

1. Sun, C. Luo, X. Zhang, M. Guo, M. Sun, H. Yu, Q. Chen, W. Yang, M. Wang, S. Zuo, P. Chen, Q. Kan, H. Zhang, Y. Wang, Z. He and J. Sun, *Nat. Commun.*, 2019, **10**, 1–10.
2. Sakavuyi and K. S. Petersen, *Tetrahedron Lett.*, 2013, **54**, 6129–6132.
3. Shiraki, K. Tsuruta, J. Morimoto, C. Ohba, A. Kawamura, R. Yoshida, R. Kawano, T. Uragami and T. Miyata, *Macromol. Rapid Commun.*, 2015, **36**, 515–519.
4. D. Newton and M. D. Pluth, *Chem. Sci.*, 2019, **10**, 10723–10727.

XI. NMR Spectra

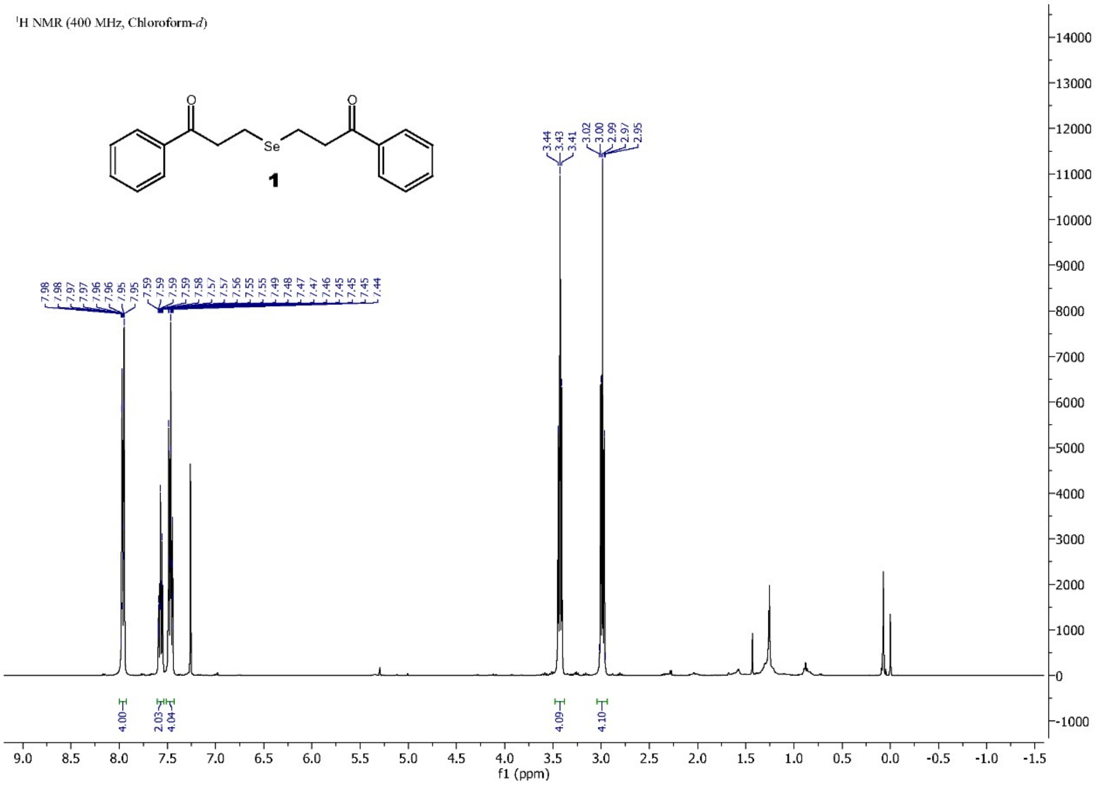

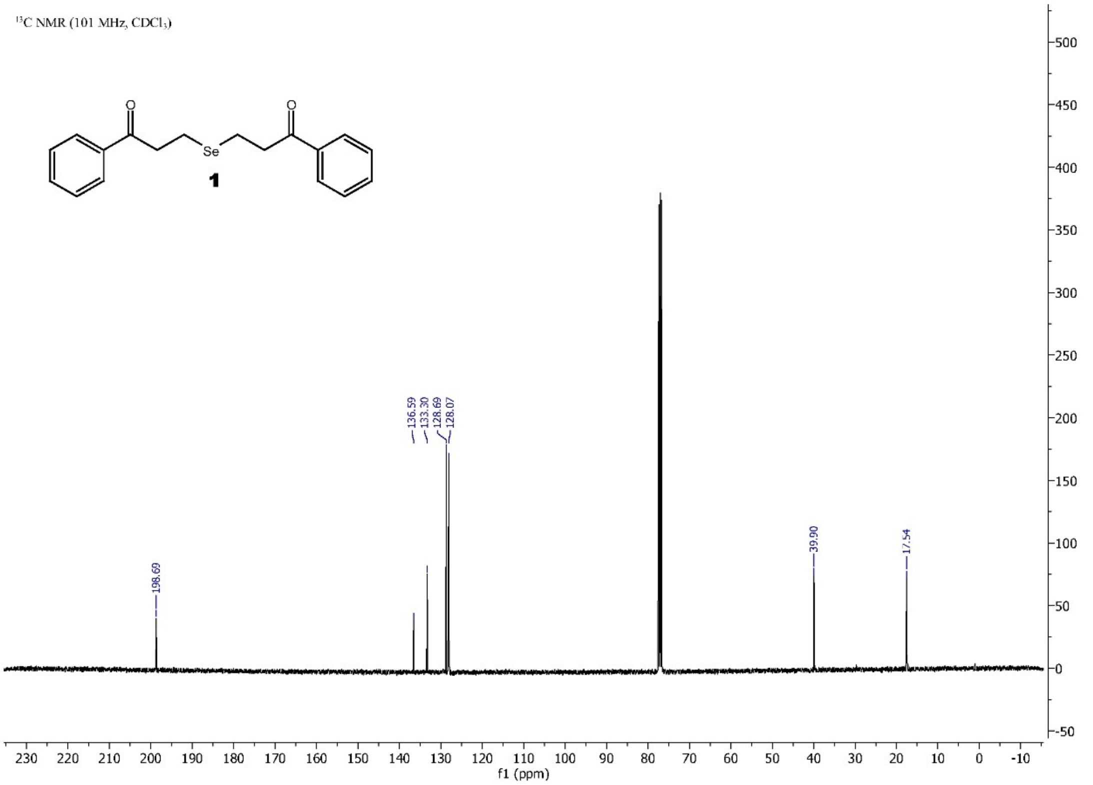

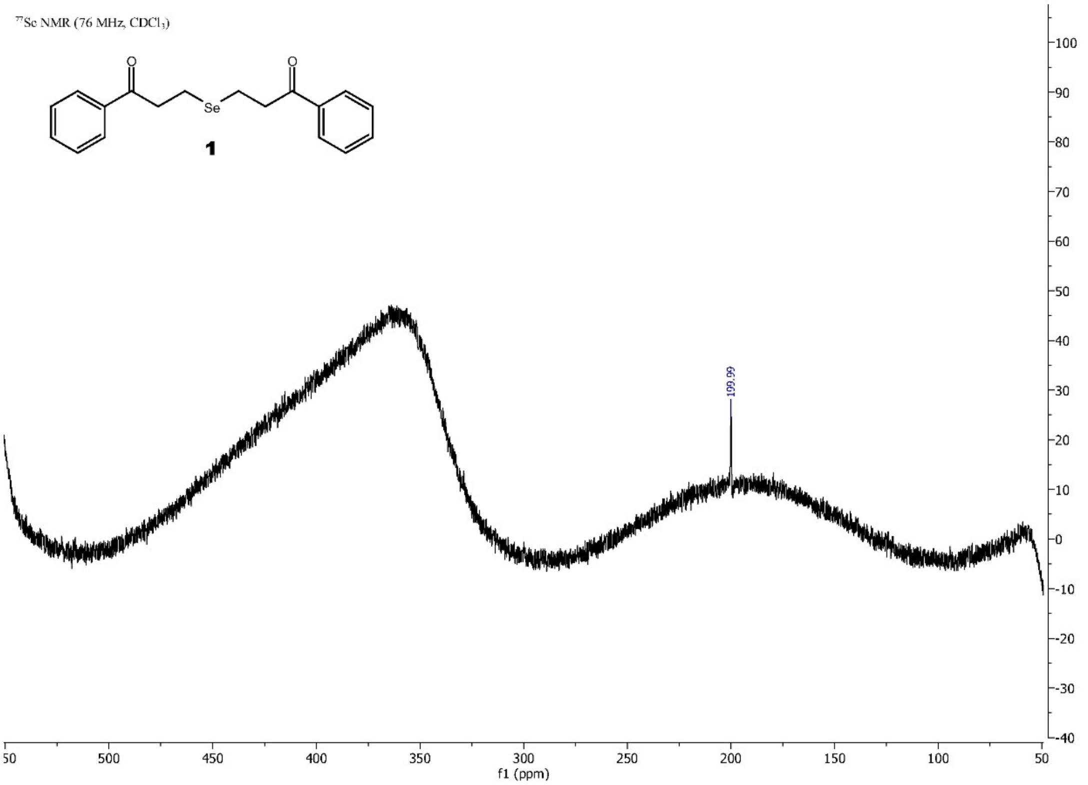
77Se NMR (76 MHz, CDCl3)
Se
1
199.99
f1 (ppm)

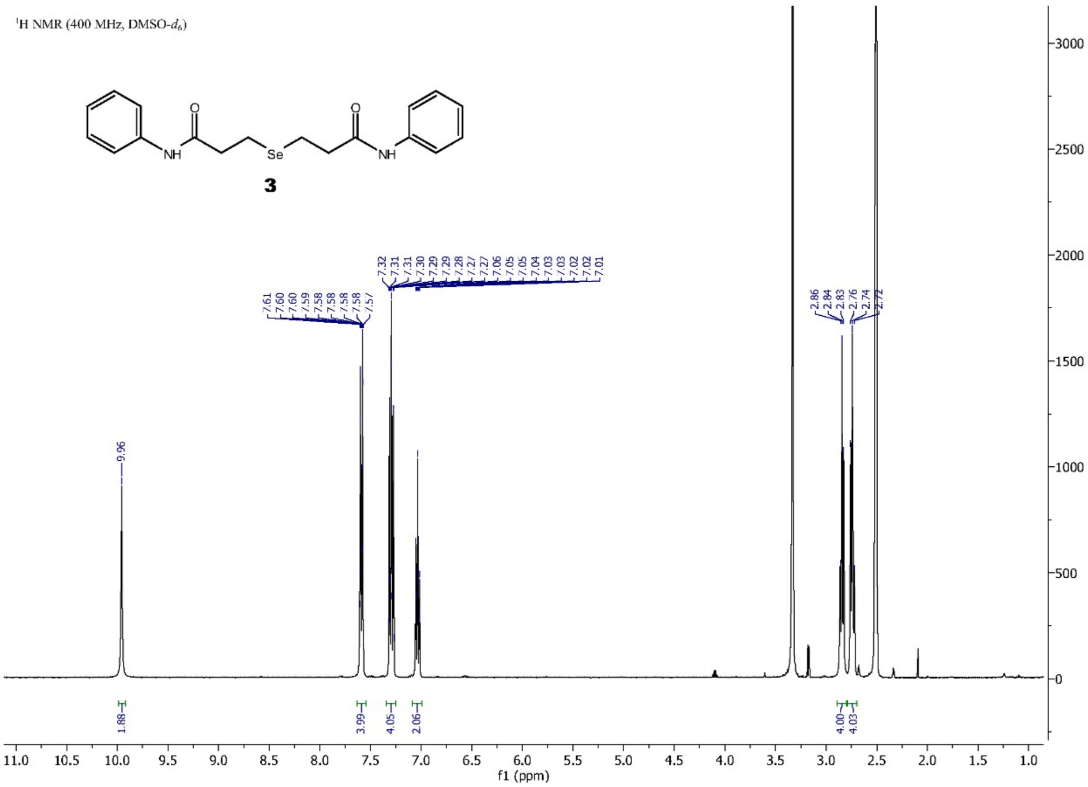
1H NMR (400 MHz, DMSO-d6)
NH
Se
NH
3
9.96
1.88
3.99
4.05
2.06
4.00
4.03
f1 (ppm)

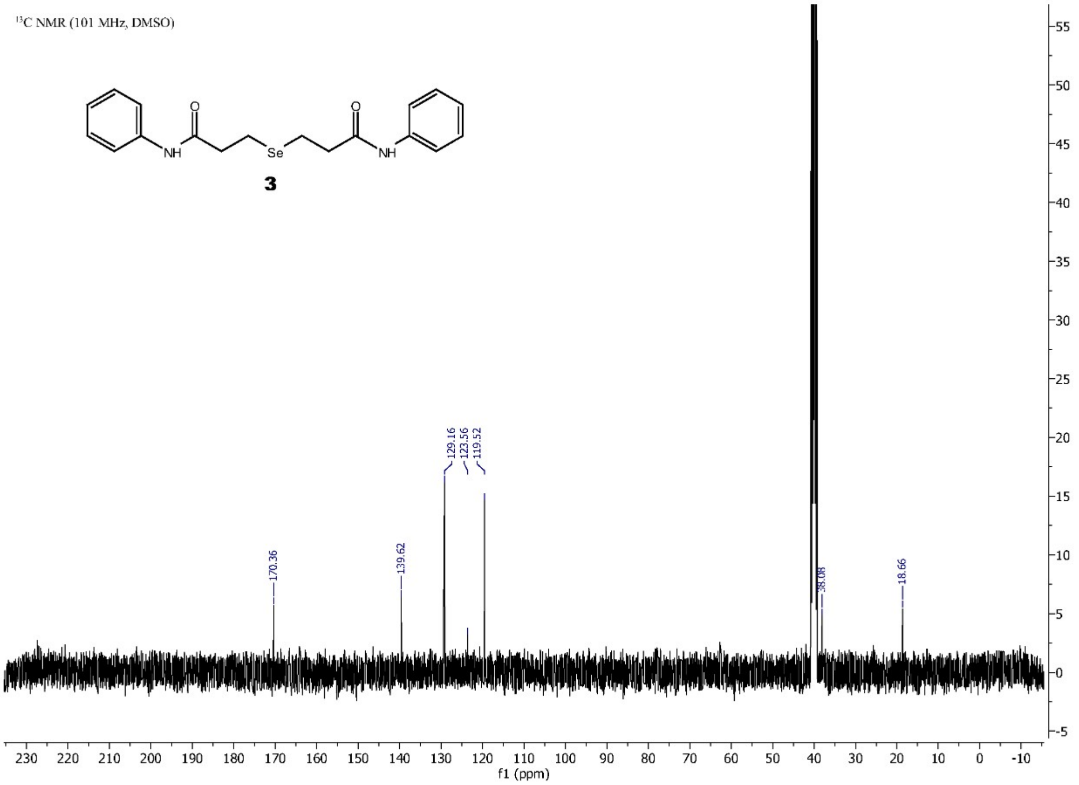

13C NMR (101 MHz, DMSO)
NH
Se
NH
O
O
3
170.36
139.62
129.16
123.56
119.52
38.08
18.66
f1 (ppm)

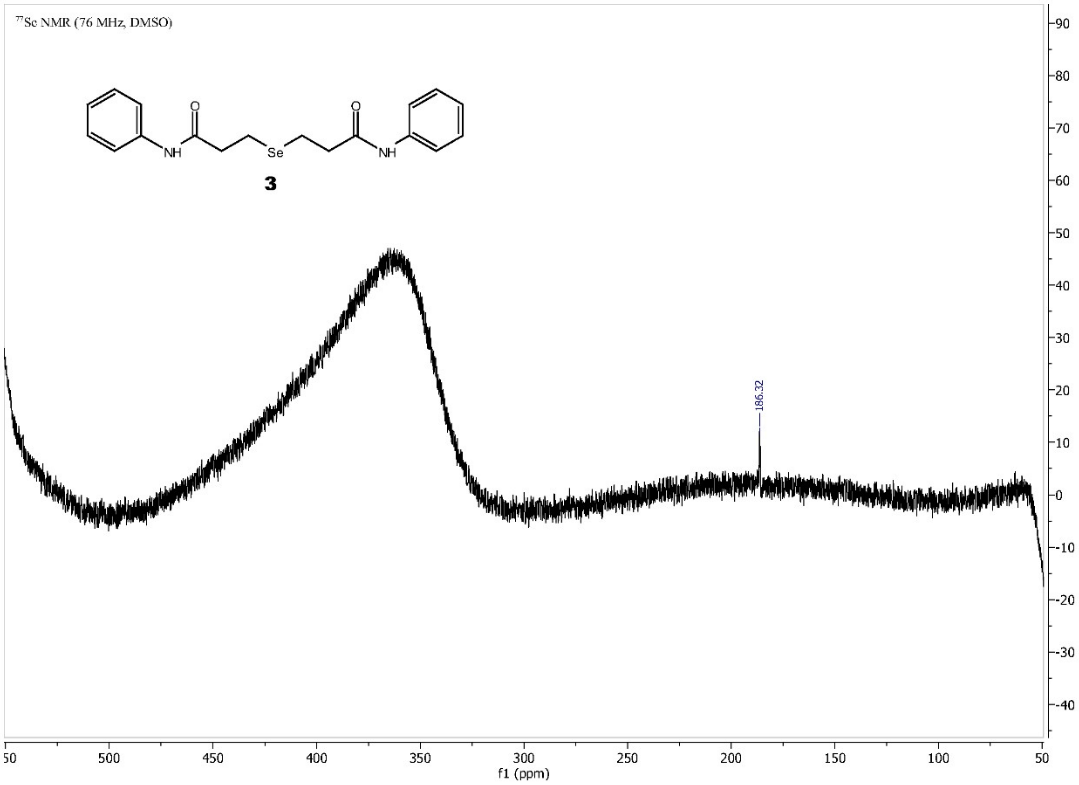

77Se NMR (76 MHz, DMSO)
NH
Se
NH
O
O
3
186.32
f1 (ppm)

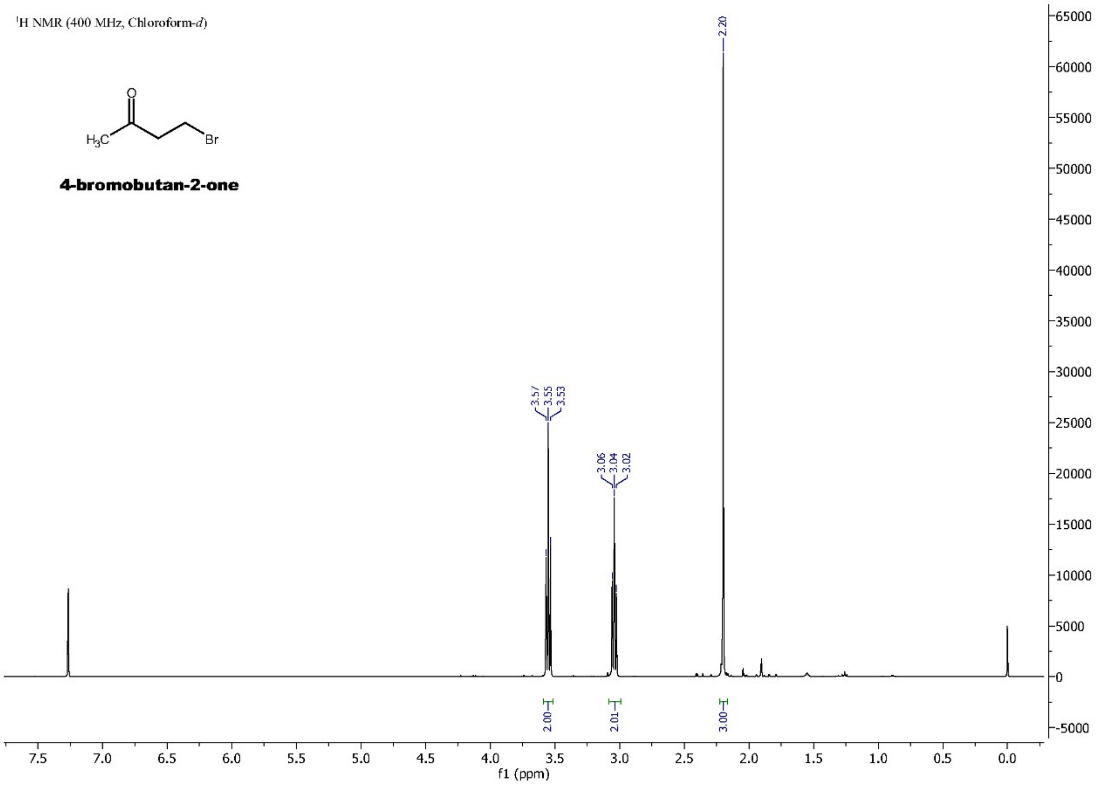
^{1}H NMR (400 MHz, Chloroform-d)
H3C
O
Br
4-bromobutan-2-one
3.57
3.55
3.53
3.06
3.04
3.02
2.20
2.00
2.01
3.00
f1 (ppm)

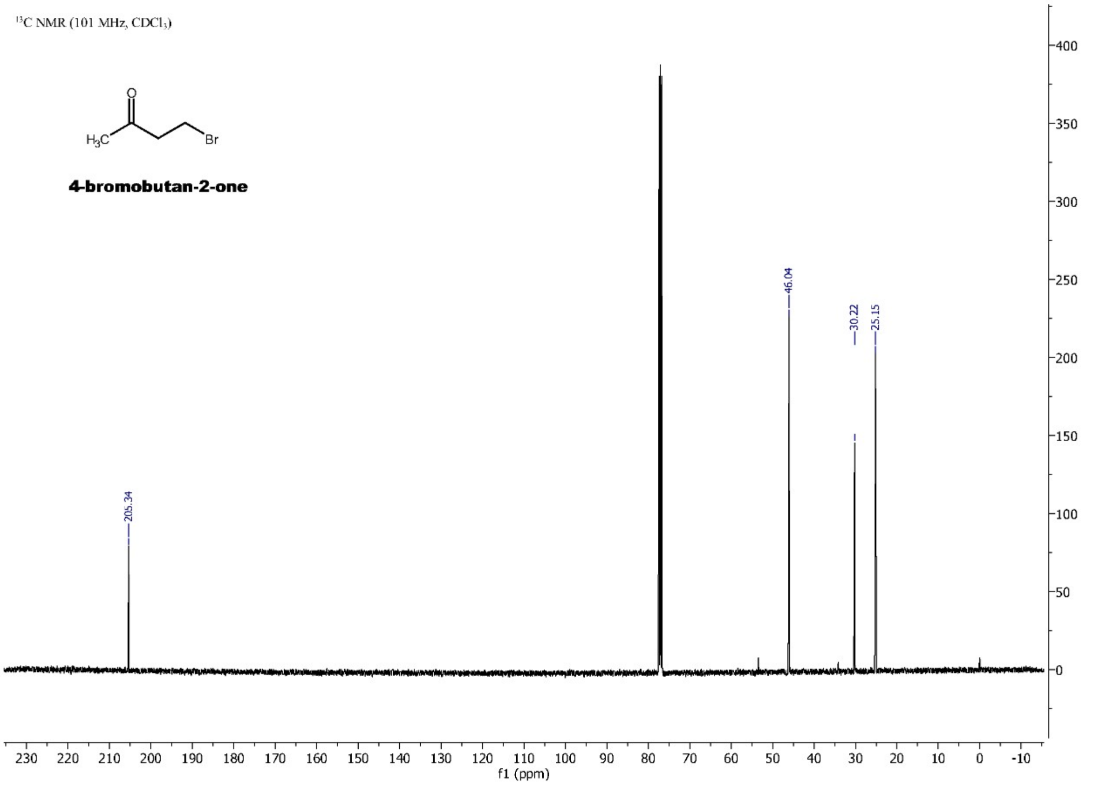
^{13}C NMR (101 MHz, CDCl3)
H3C
O
Br
4-bromobutan-2-one
205.34
46.04
30.22
25.15
f1 (ppm)

^{1}H NMR (400 MHz, Chloroform-*d*)

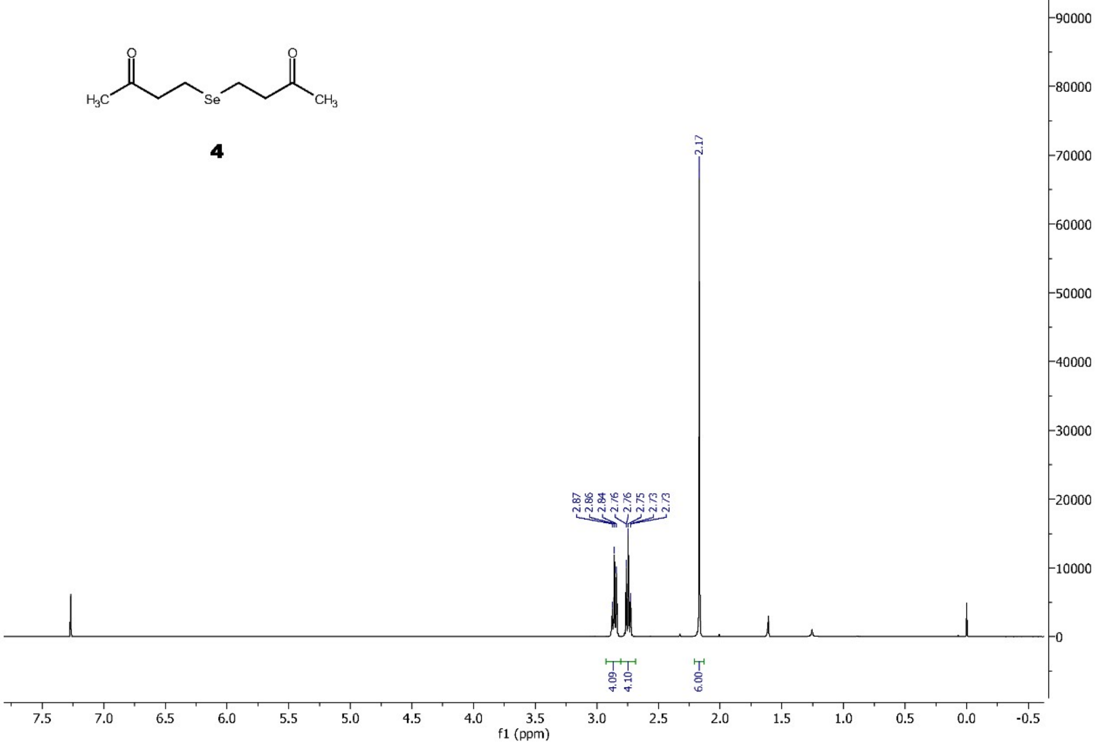

^{13}C NMR (101 MHz, $CDCl_3$)

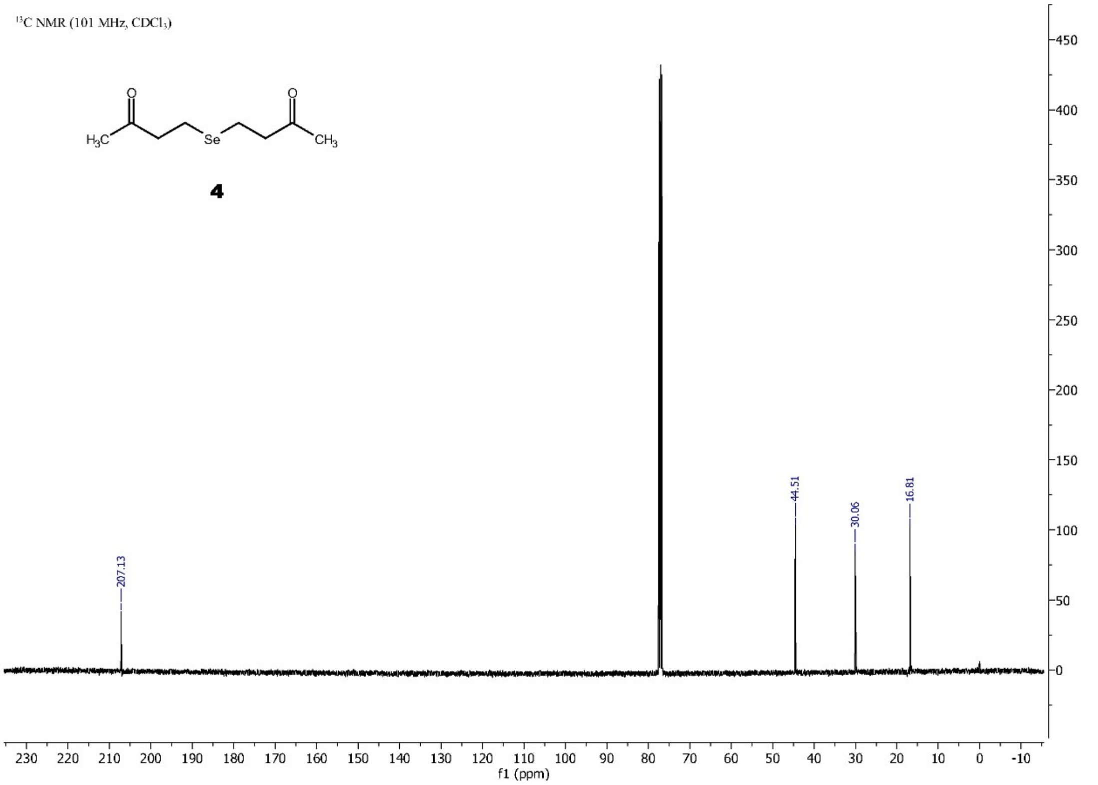

^{77}Se NMR (76 MHz, CDCl$_3$)

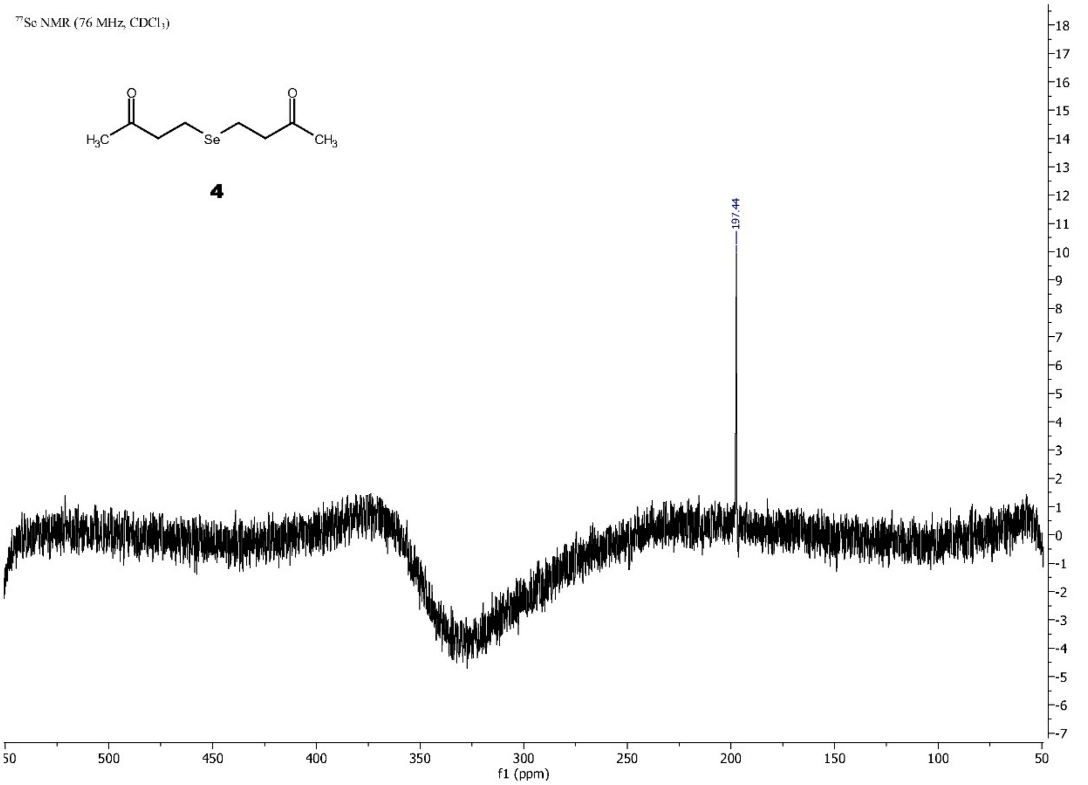

4

^{1}H NMR (400 MHz, Chloroform-*d*)

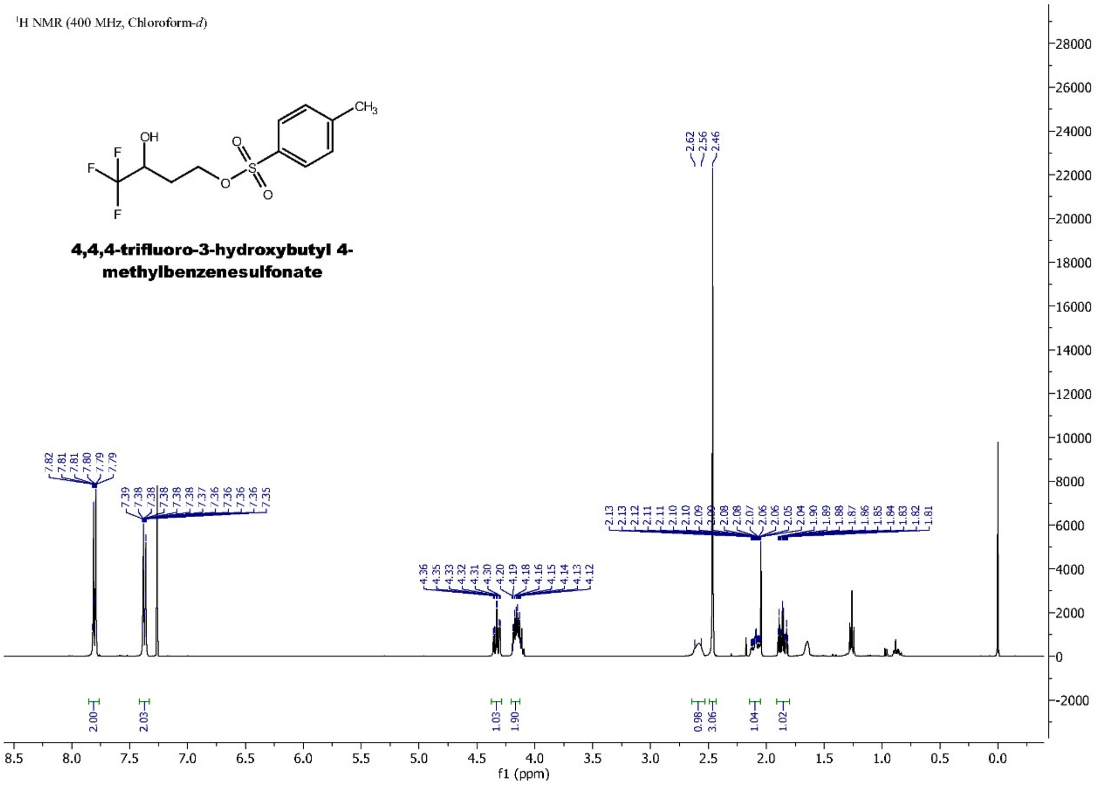

4,4,4-trifluoro-3-hydroxybutyl 4-methylbenzenesulfonate

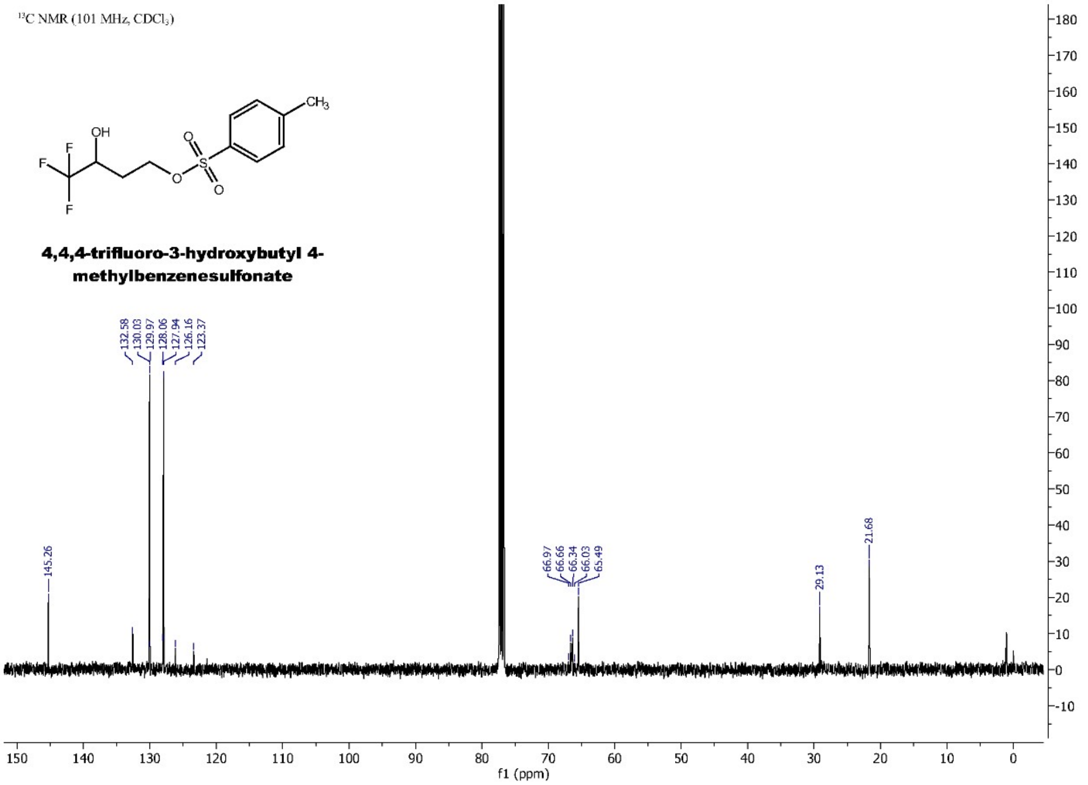

13C NMR (101 MHz, CDCl3)
4,4,4-trifluoro-3-hydroxybutyl 4-methylbenzenesulfonate
132.58
130.03
129.97
128.06
127.94
126.16
123.37
145.26
66.97
66.66
66.34
66.03
65.49
29.13
21.68
f1 (ppm)

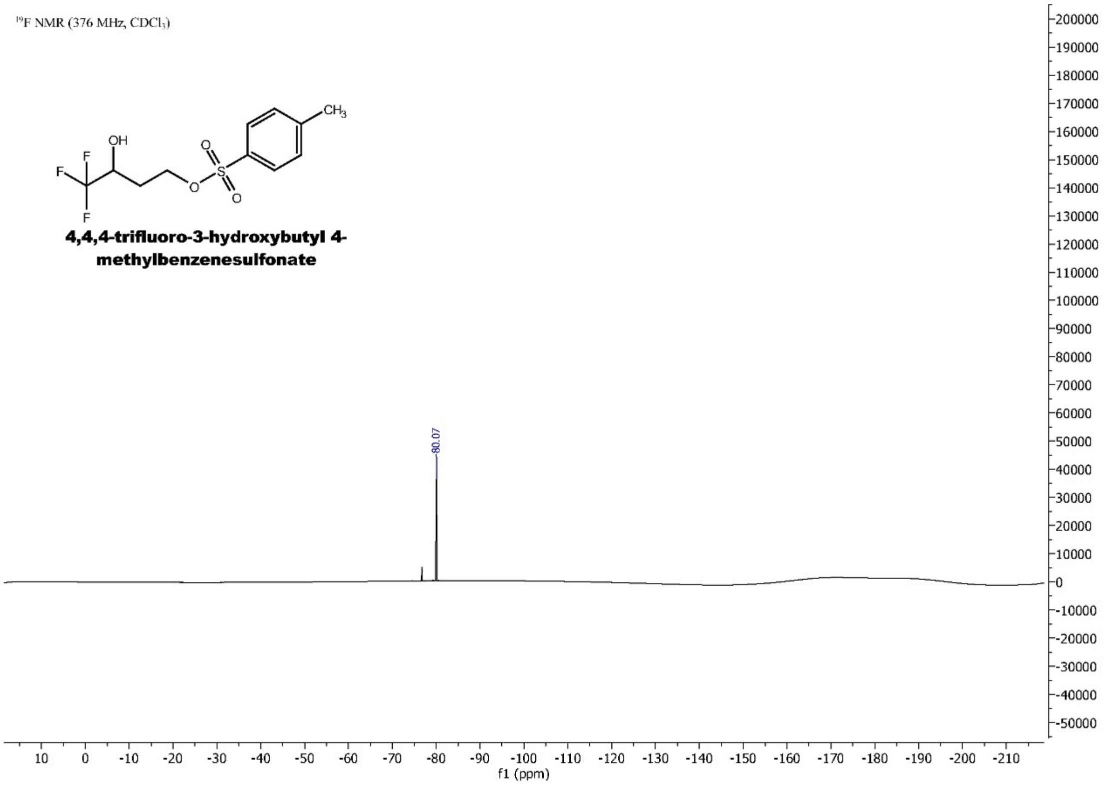

19F NMR (376 MHz, CDCl3)
4,4,4-trifluoro-3-hydroxybutyl 4-methylbenzenesulfonate
80.07
f1 (ppm)

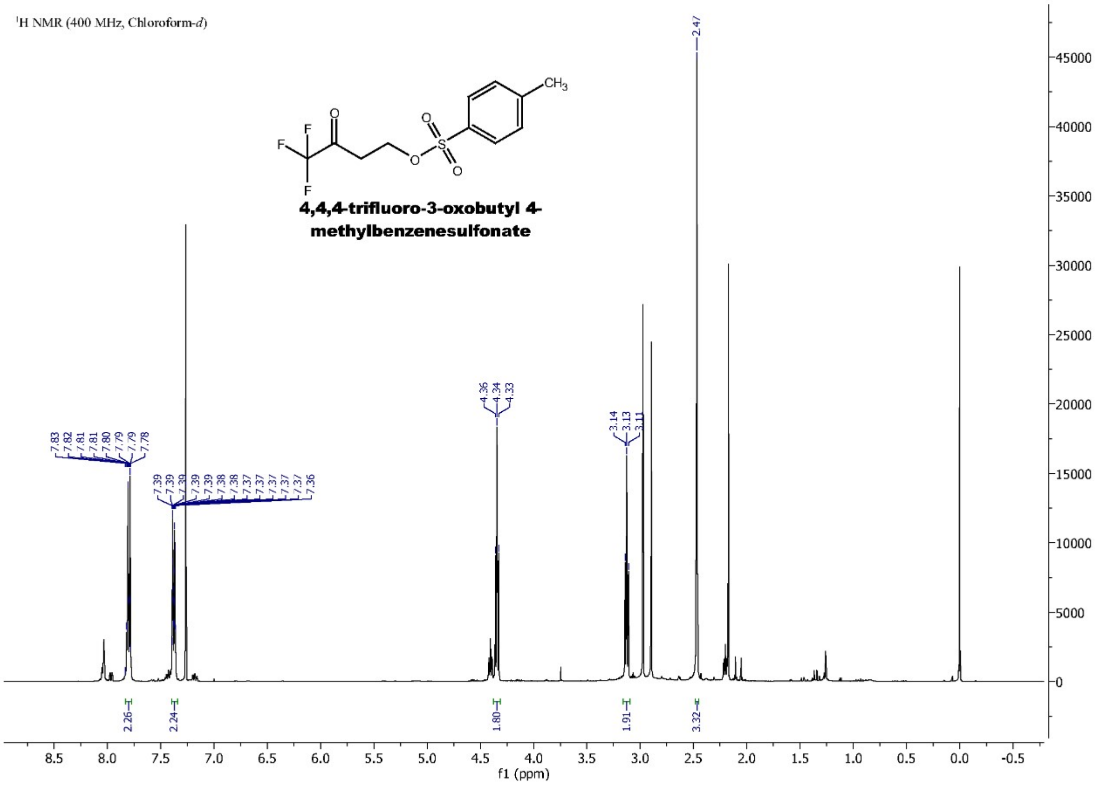
^{1}H NMR (400 MHz, Chloroform-*d*)
4,4,4-trifluoro-3-oxobutyl 4-methylbenzenesulfonate
7.83 7.82 7.81 7.81 7.80 7.79 7.79 7.78
7.39 7.39 7.39 7.39 7.39 7.38 7.38 7.37 7.37 7.37 7.37 7.37 7.36
4.36 4.34 4.33
3.14 3.13 3.11
2.47
2.26 2.24 1.80 1.91 3.32
f1 (ppm)

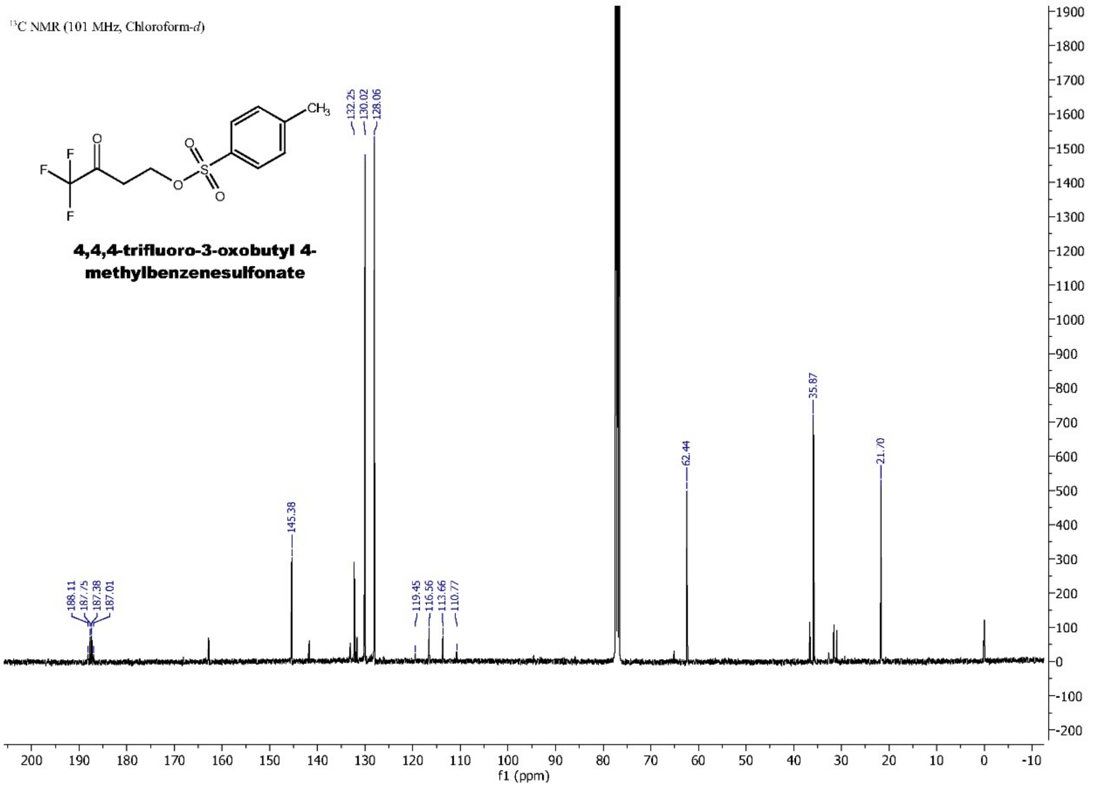
^{13}C NMR (101 MHz, Chloroform-*d*)
4,4,4-trifluoro-3-oxobutyl 4-methylbenzenesulfonate
188.11 187.75 187.38 187.01
145.38
132.25 130.02 128.06
119.45 116.56 113.66 110.77
62.44
35.87
21.70
f1 (ppm)

^{19}F NMR (376 MHz, CDCl$_3$)

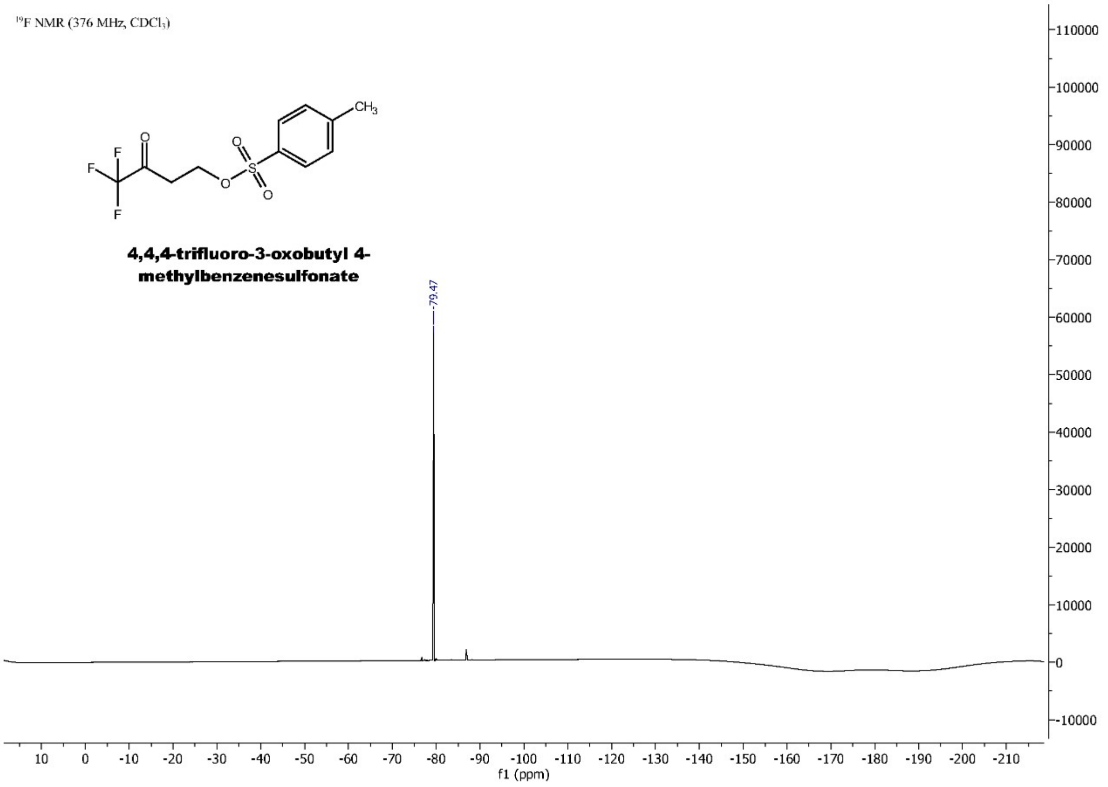

^{1}H NMR (400 MHz, Chloroform-*d*)

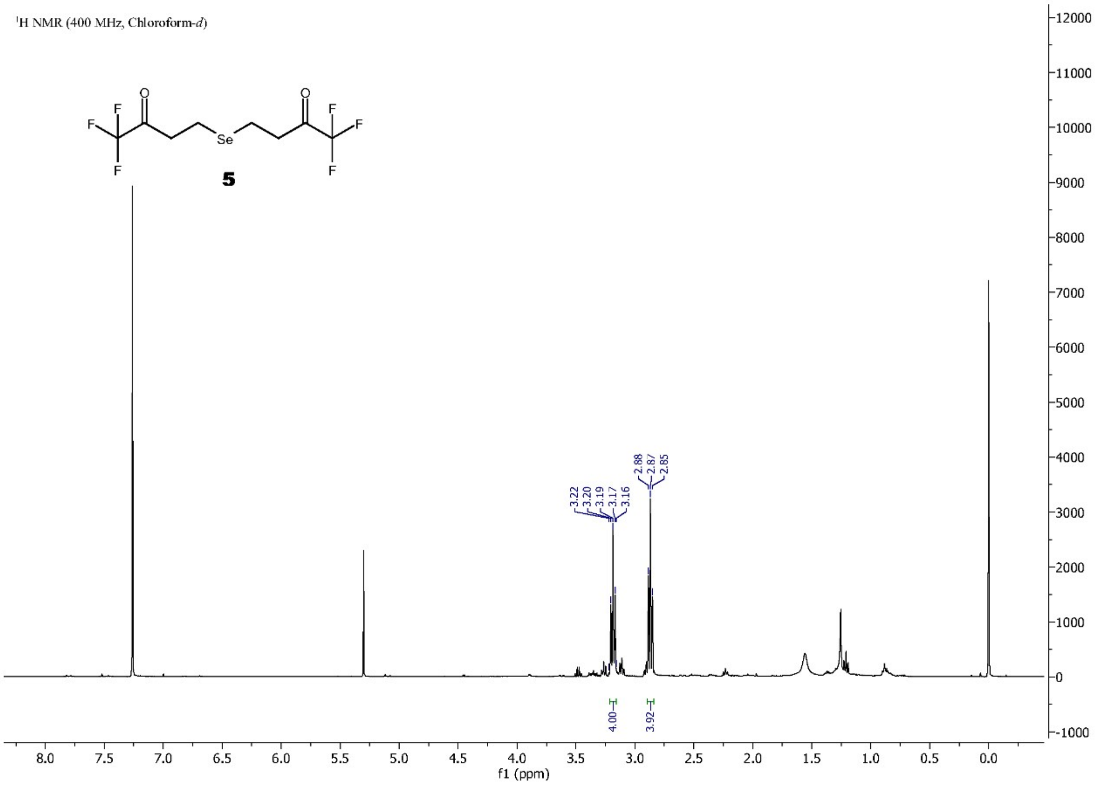

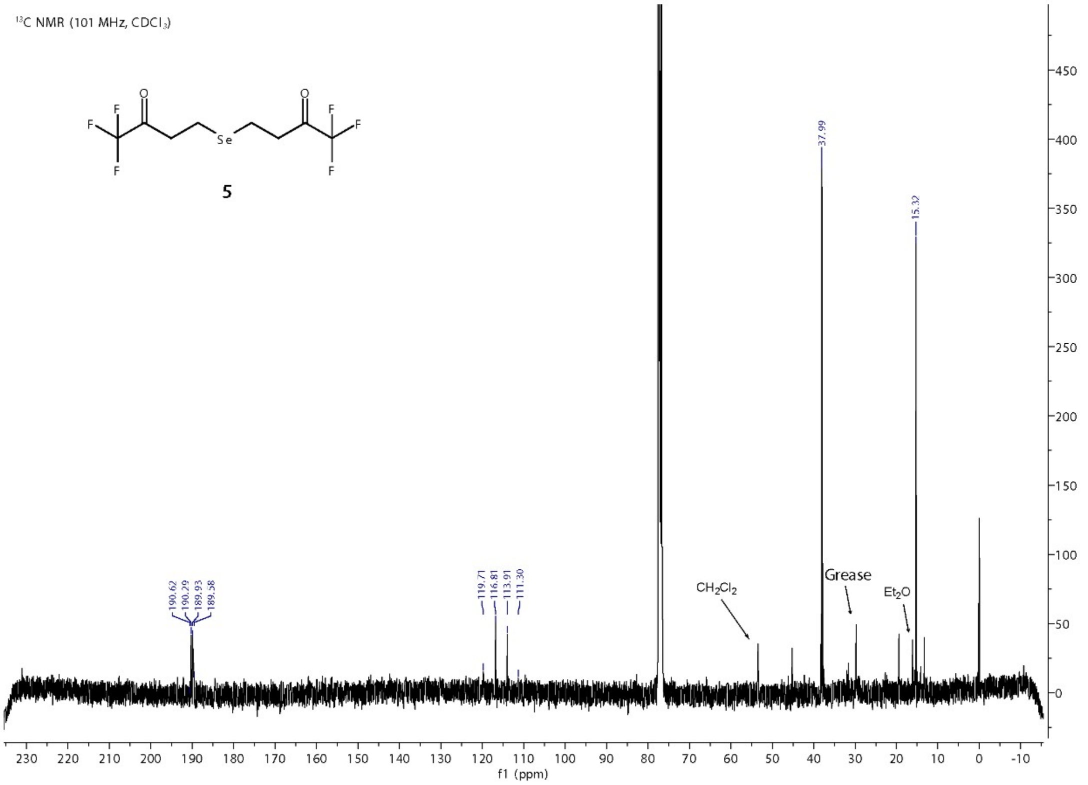

13C NMR (101 MHz, CDCl3)
5
190.62
190.29
189.93
189.58
119.71
116.81
113.91
111.30
37.99
15.32
CH2Cl2
Grease
Et2O
f1 (ppm)

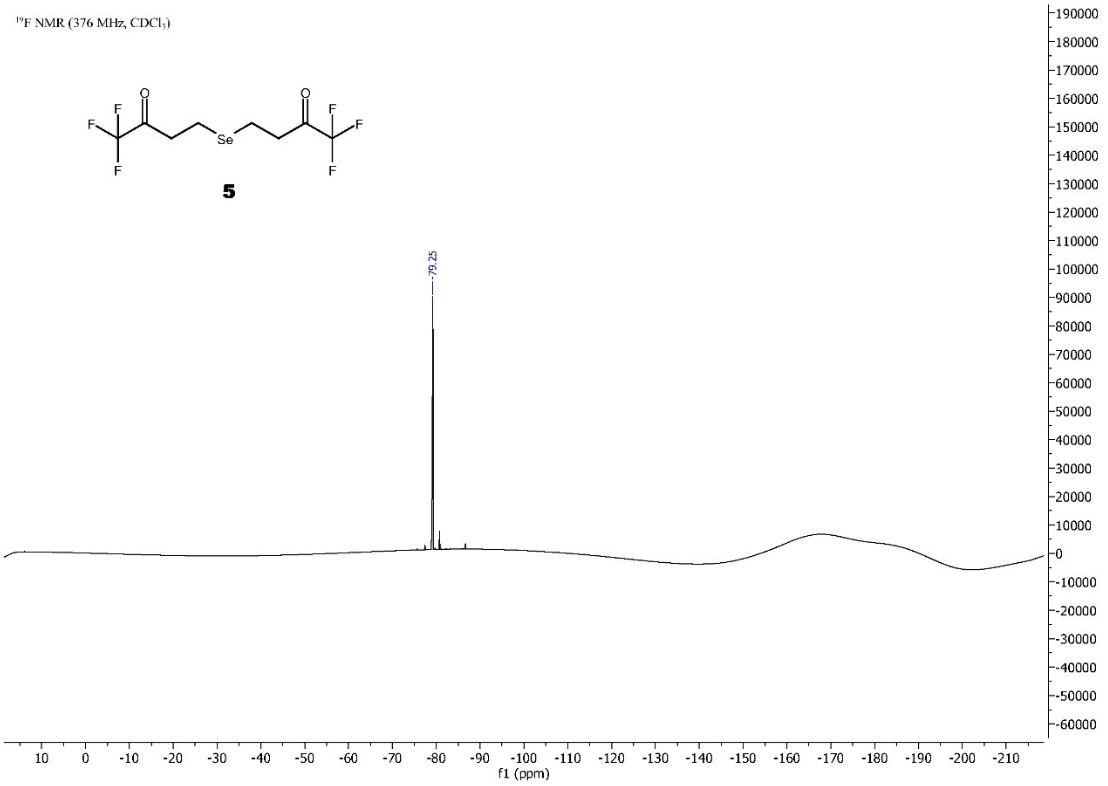

19F NMR (376 MHz, CDCl3)
5
-79.25
f1 (ppm)

^{77}Se NMR (76 MHz, CDCl$_3$)

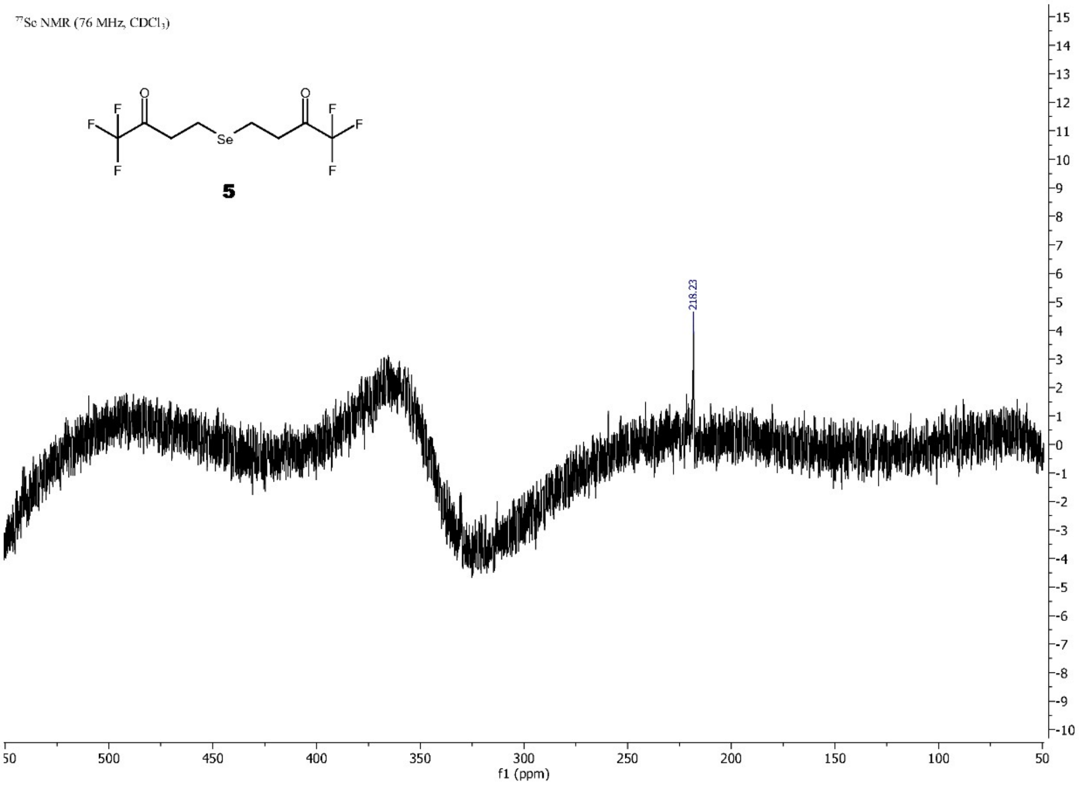

^{1}H NMR (400 MHz, Chloroform-d)

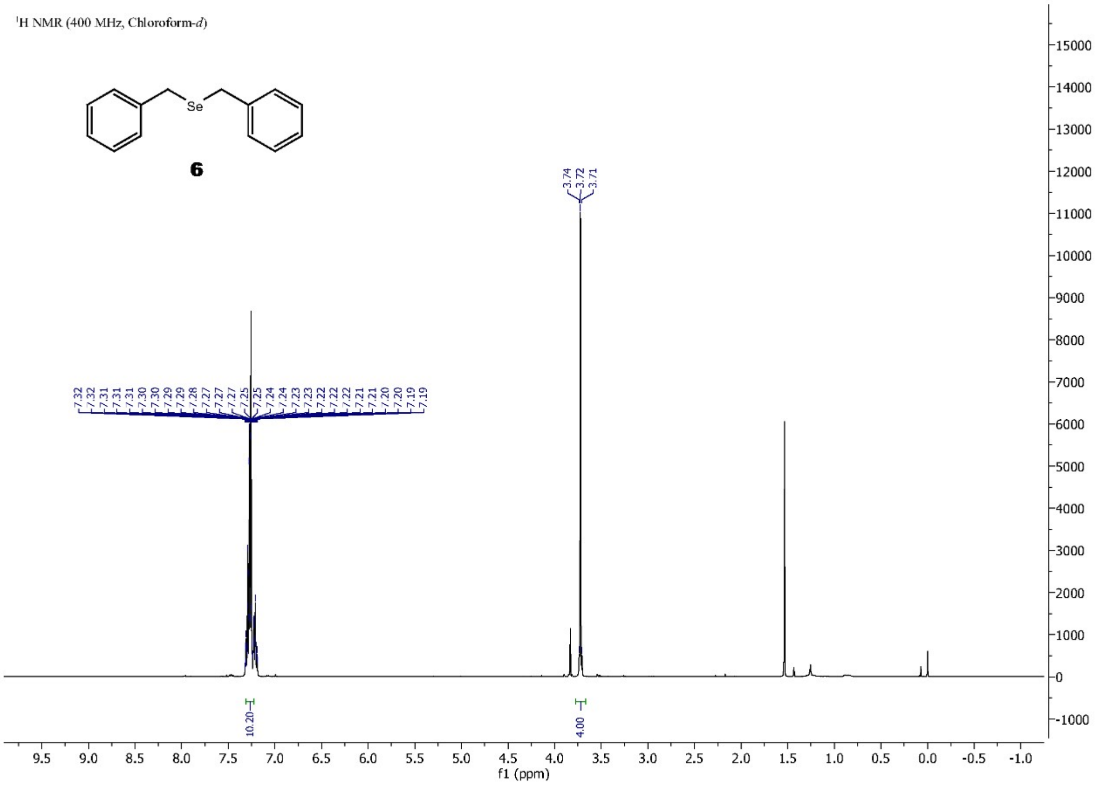

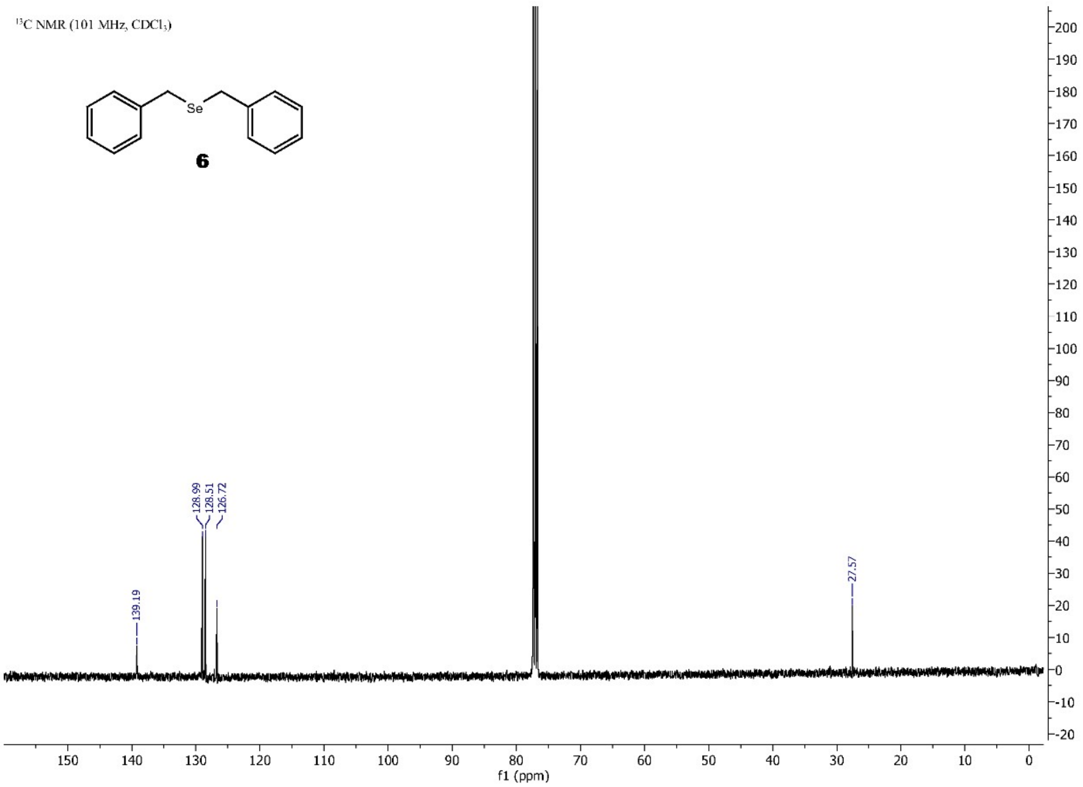

^{13}C NMR (101 MHz, CDCl$_3$)
Se
6
139.19
128.99
128.51
126.72
27.57
f1 (ppm)

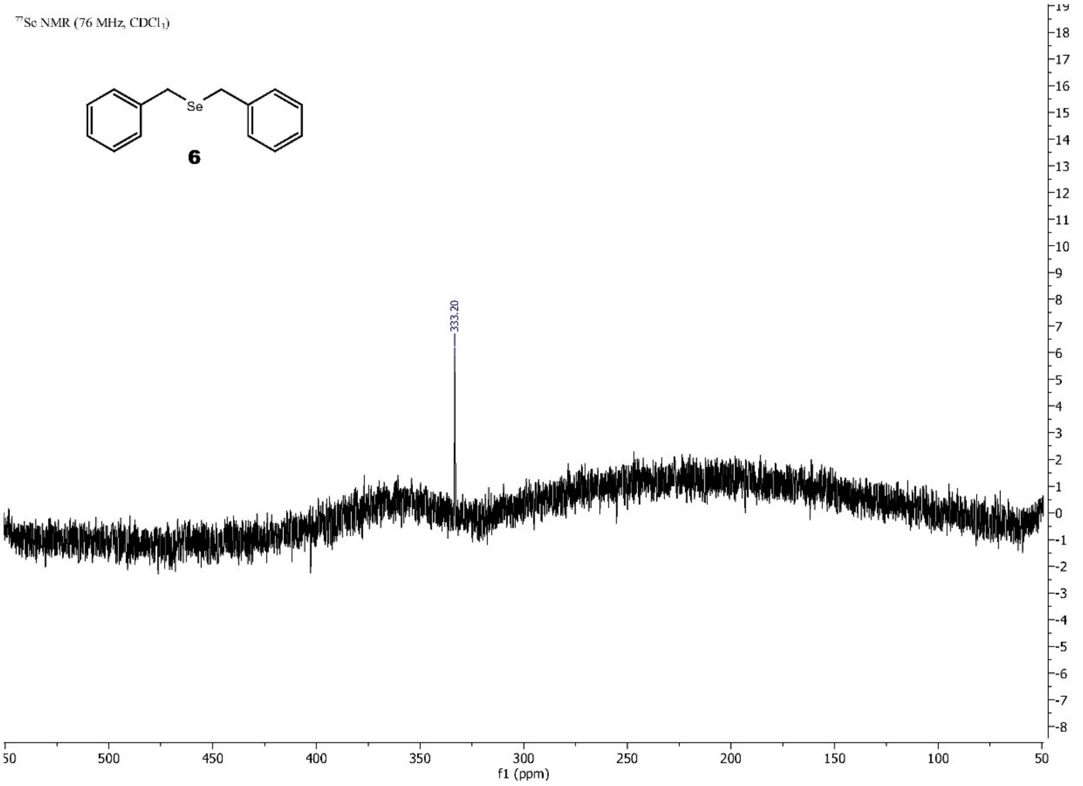

^{77}Se NMR (76 MHz, CDCl$_3$)
Se
6
333.20
f1 (ppm)

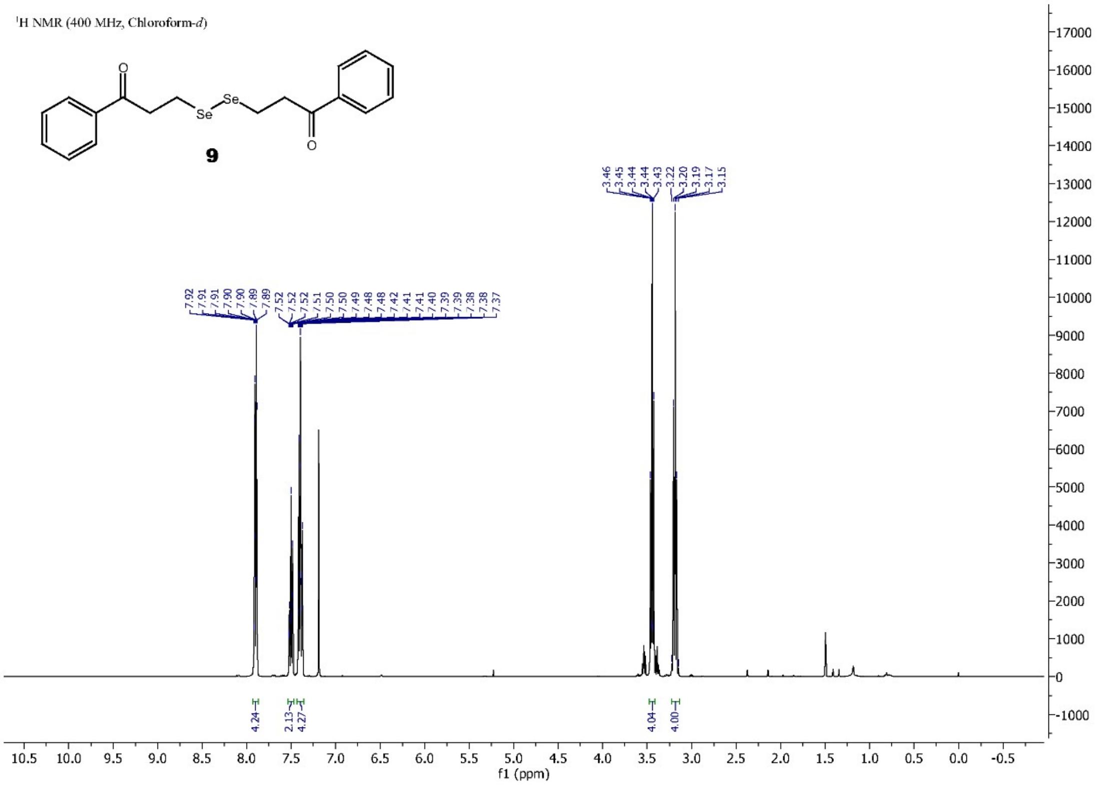
1H NMR (400 MHz, Chloroform-d)
9
7.92 7.91 7.91 7.90 7.90 7.89 7.89 7.52 7.52 7.52 7.51 7.50 7.50 7.49 7.48 7.48 7.42 7.41 7.41 7.40 7.39 7.39 7.38 7.38 7.37
3.46 3.45 3.44 3.44 3.43 3.22 3.20 3.19 3.17 3.15
4.24 2.13 4.27 4.04 4.00
f1 (ppm)

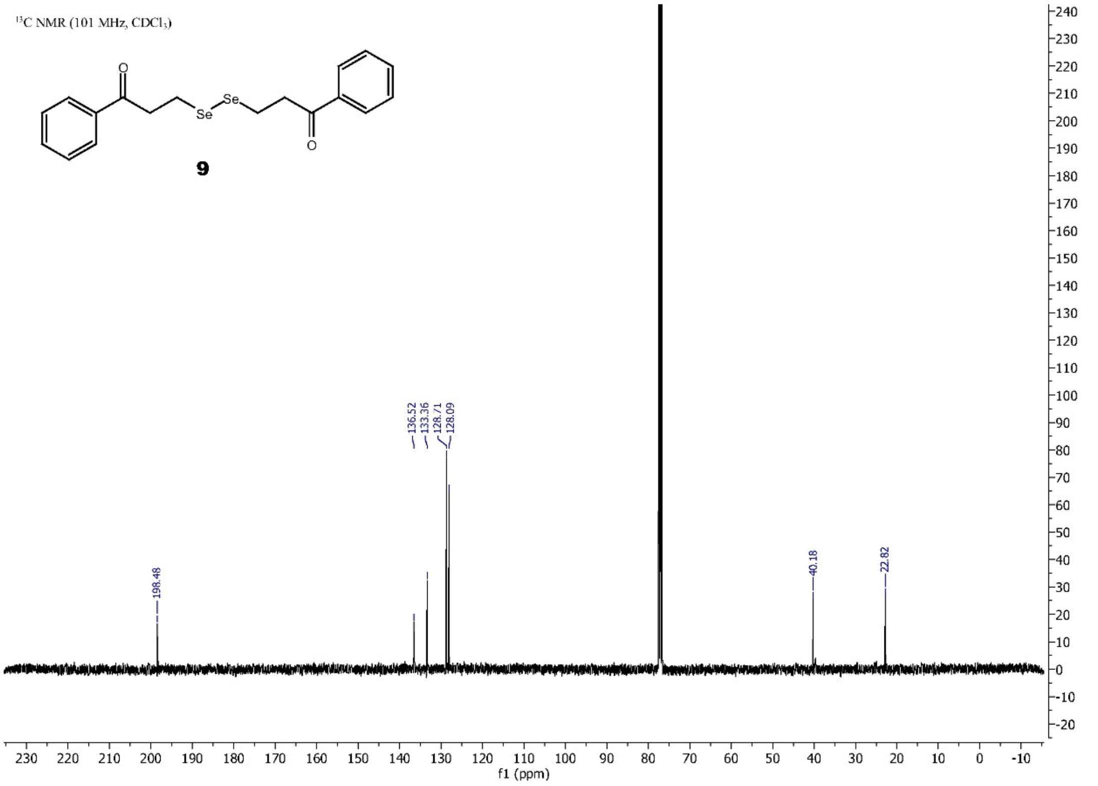
13C NMR (101 MHz, CDCl3)
9
198.48
136.52 133.36 128.71 128.09
40.18
22.82
f1 (ppm)

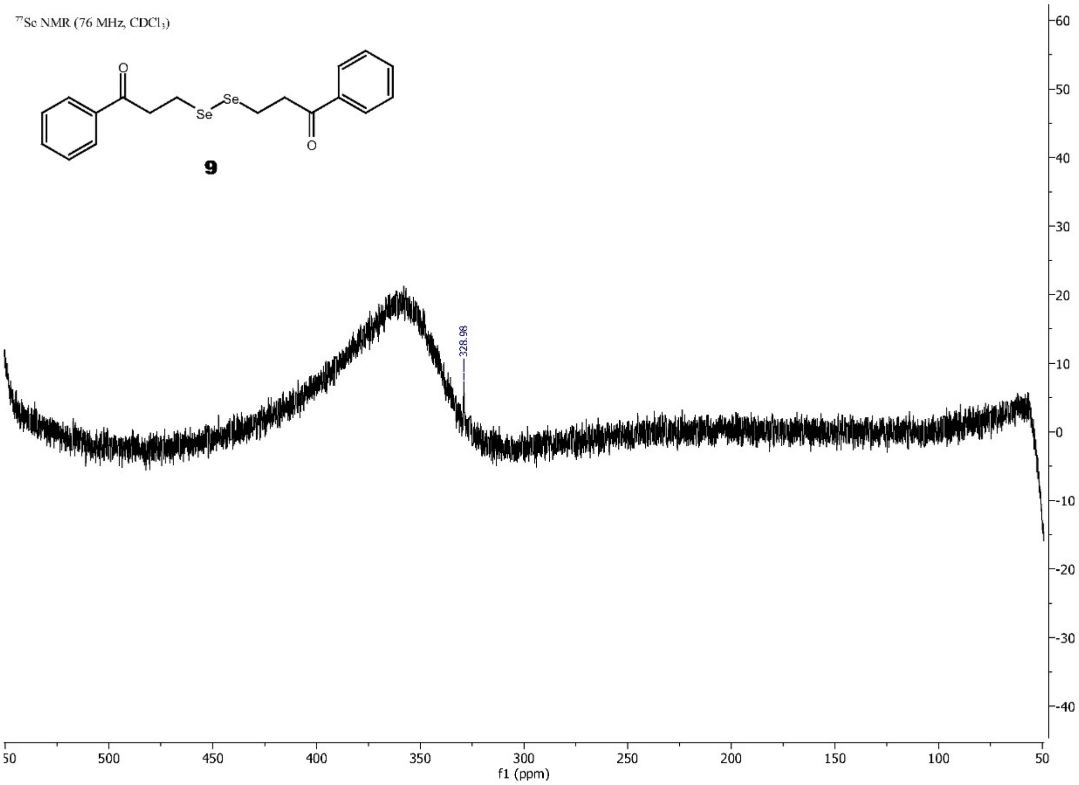

77Se NMR (76 MHz, CDCl3)
9
328.98
f1 (ppm)

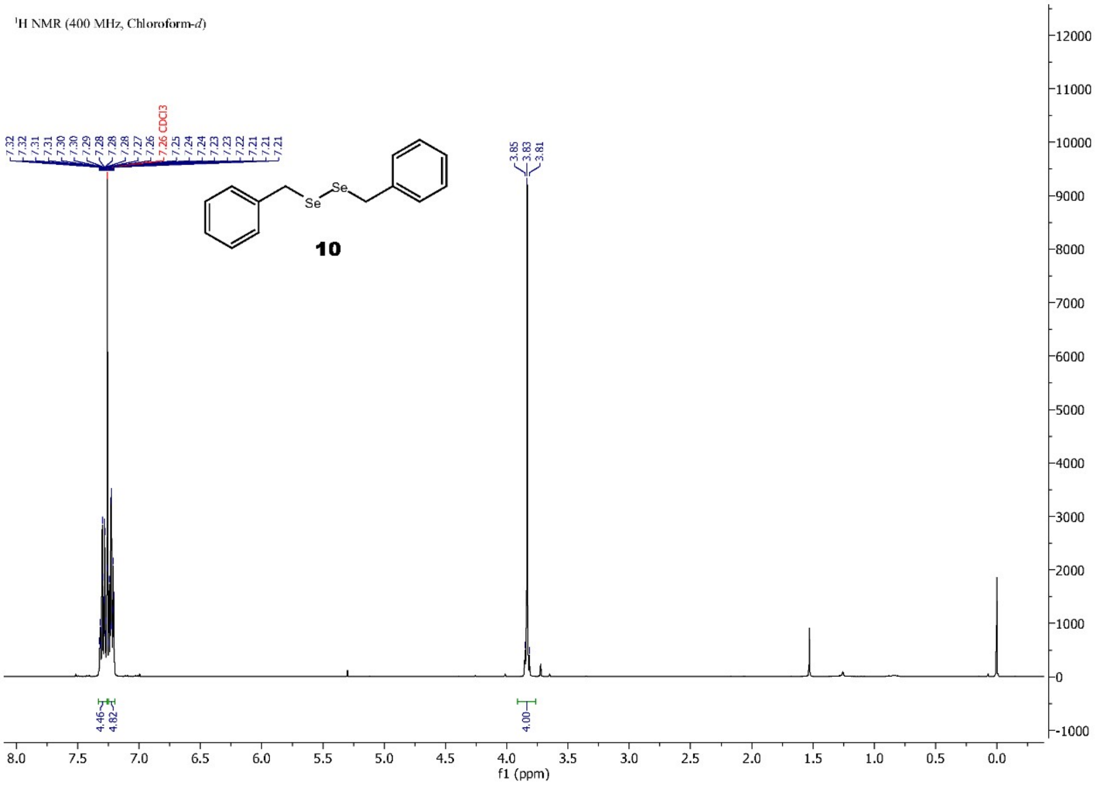

1H NMR (400 MHz, Chloroform-d)
10
7.26 CDCl3
3.85
3.83
3.81
4.46
4.82
4.00
f1 (ppm)

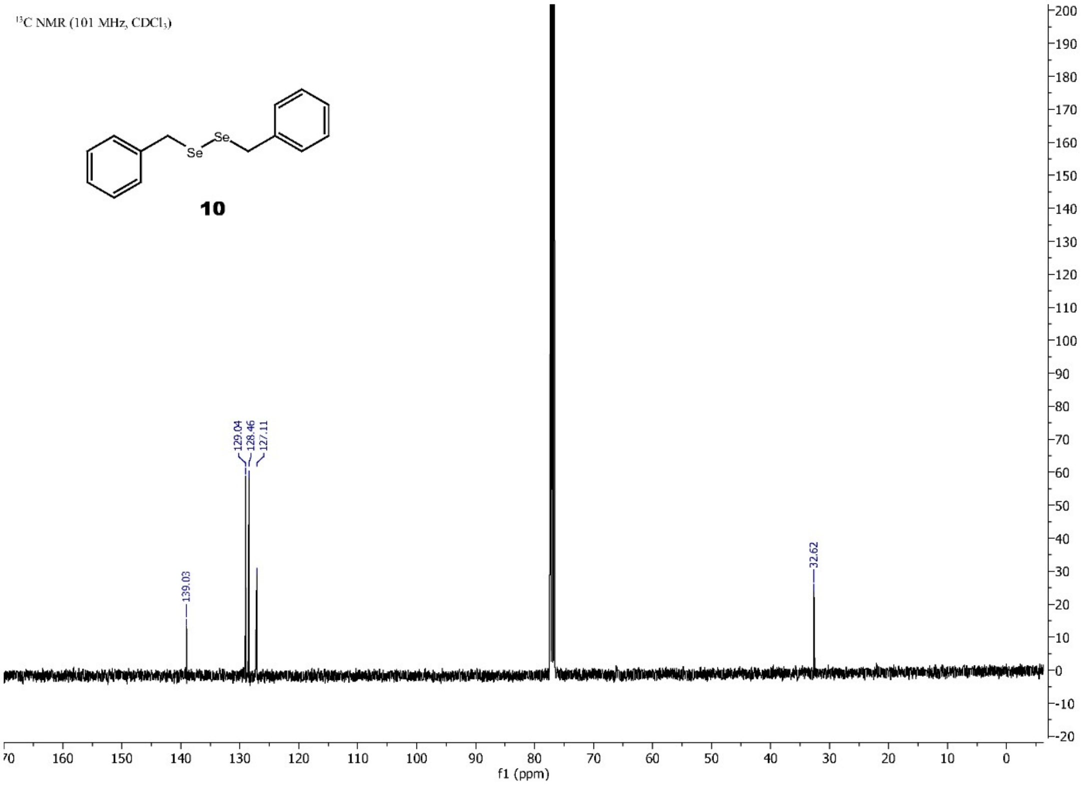
^{13}C NMR (101 MHz, $CDCl_3$)
Se
Se
10
139.03
129.04
128.46
127.11
32.62
f1 (ppm)

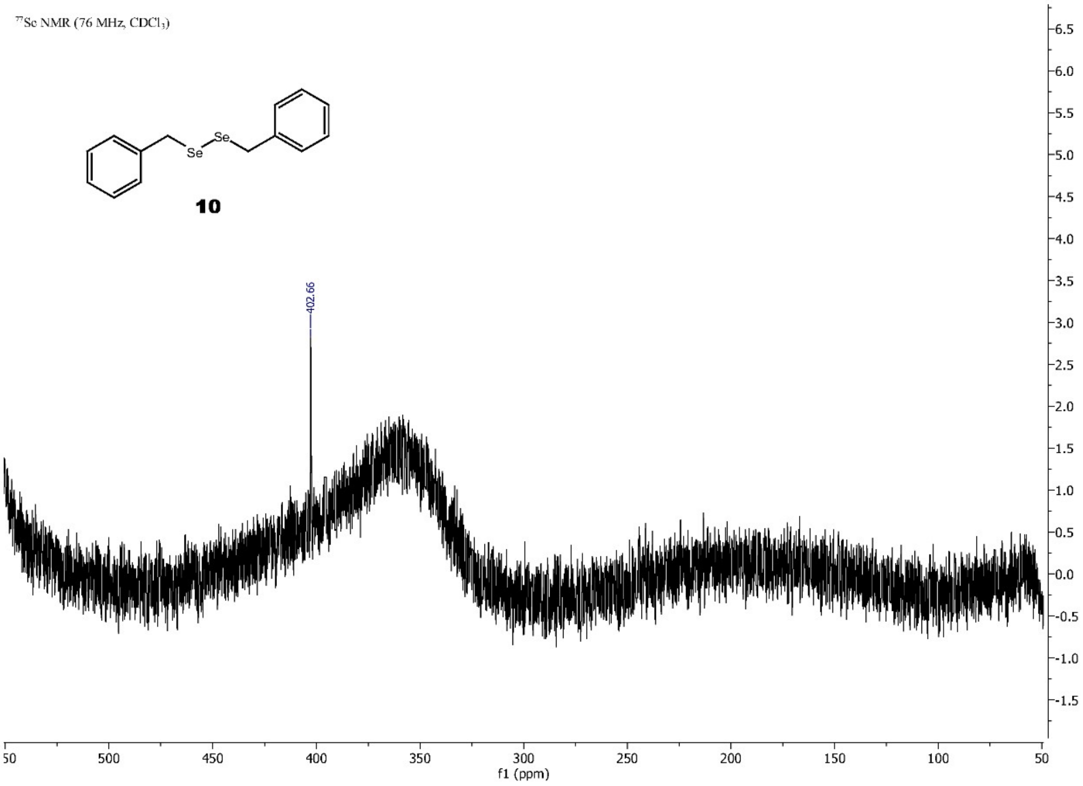
^{77}Se NMR (76 MHz, $CDCl_3$)
Se
Se
10
402.66
f1 (ppm)

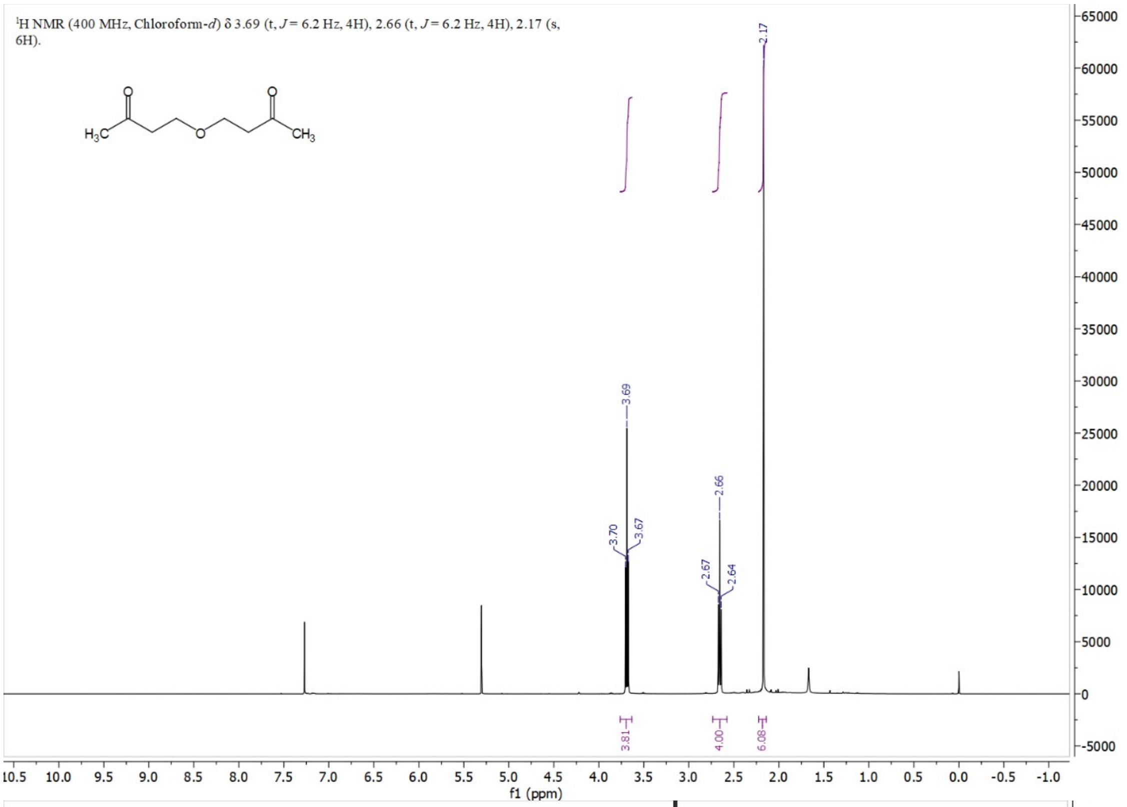

^{1}H NMR (400 MHz, Chloroform-d) δ 3.69 (t, J = 6.2 Hz, 4H), 2.66 (t, J = 6.2 Hz, 4H), 2.17 (s, 6H).
3.69
3.70
3.67
2.66
2.67
2.64
2.17
3.81
4.00
6.08
f1 (ppm)

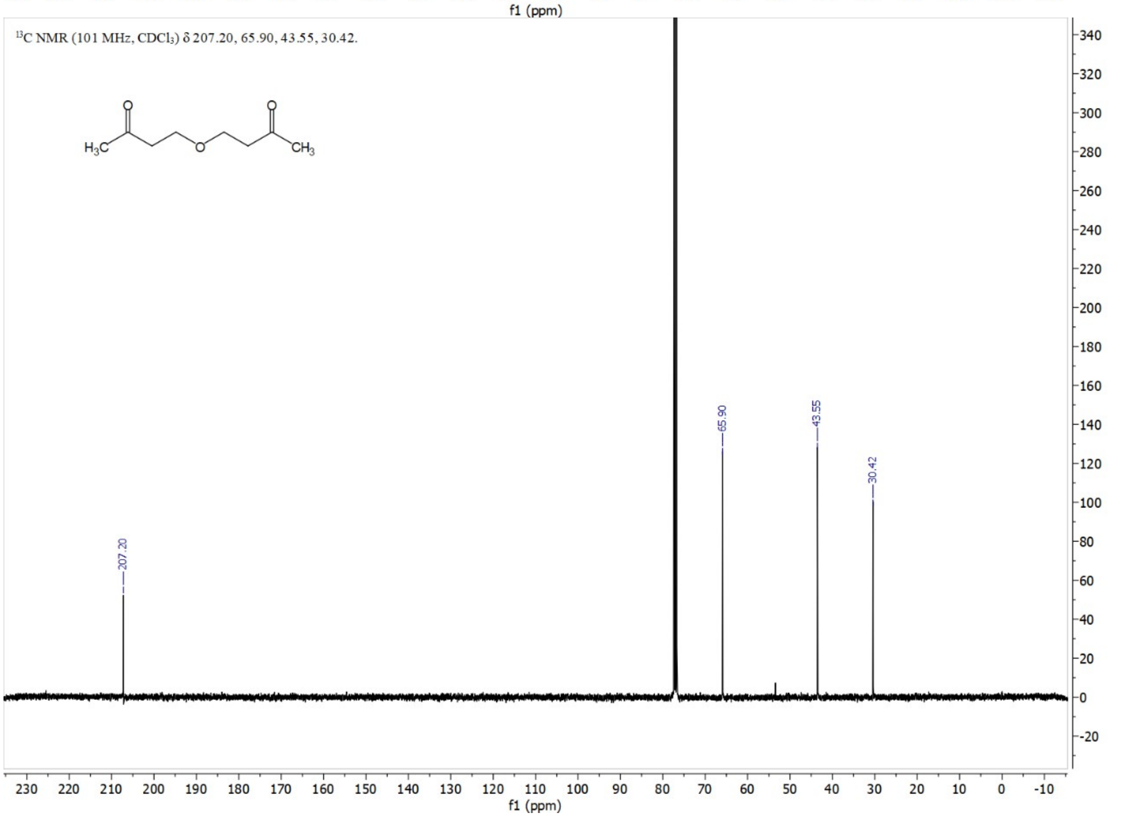

^{13}C NMR (101 MHz, CDCl3) δ 207.20, 65.90, 43.55, 30.42.
207.20
65.90
43.55
30.42
f1 (ppm)
